# Global youth vaping and respiratory health: *epidemiology, interventions, and policies*

**DOI:** 10.1038/s41533-022-00277-9

**Published:** 2022-04-11

**Authors:** Lynnette Nathalie Lyzwinski, John A. Naslund, Christopher J. Miller, Mark J. Eisenberg

**Affiliations:** 1 Center for Clinical Epidemiology, Lady Davis Institute, Jewish General Hospital, Montreal, QC Canada; 2 Department of Medicine, McGill University, Montreal, QC Canada; 3 Global Health and Social Medicine, Harvard Medical School, Boston, MA USA; 4 The Center for Healthcare Organization and Implementation Research (CHOIR) at the VA Boston Healthcare System, Boston, MA USA; 5 Department of Psychiatry, Harvard Medical School, Harvard University, Boston, MA USA; 6 Division of Cardiology, Jewish General Hospital, McGill University, Montreal, QC Canada; 7 Departments of Medicine and of Epidemiology, Biostatistics and Occupational Health, McGill University, Montreal, QC Canada

**Keywords:** Epidemiology, Respiratory signs and symptoms, Epidemiology, Health policy, Disease prevention

## Abstract

E-cigarette usage (also known as e-cigarettes or vaping products) has increasingly been recognized as a global public health problem. One challenge in particular involves their marketing to minors (teenagers and children) and the rising prevalence of use in this population. E-cigarettes unnecessarily expose minors to health risks, these include respiratory health problems, such as exacerbations of asthma, bronchitis, and respiratory-tract irritation. Nicotine, commonly found in e-cigarettes, is also associated with cognitive impairment and neurodevelopmental problems. E-cigarettes are also risk factors for downstream substance use, including cigarettes and cannabis initiation (the gateway hypothesis), which compounds health risks in dual users. Current public health preventative and intervention studies are limited, and there is a clear need for more interventions that may prevent usage and assist with cessation in this vulnerable population. Physician education and screening uptake should also be enhanced. Stricter public health policy and protection measures are also needed on a global scale to limit e-cigarette exposure in minors.

## Introduction

The use of electronic cigarettes (also known as e-cigarettes, e-cigs, or vaping products) has increasingly been recognized as a global public health problem^1^. Vaping consists of inhaling a smoke-free aerosol through a mouthpiece, which is produced through the heating of a liquid such as glycol or glycerin in an electronic device^2,3^. Most e-cigarettes have the shape of a pen, but others are more discrete-looking such as JUUL, which resembles a USB drive and is popular among teenagers^4^. Common terminology for e-cigarettes is summarized in Table 1. E-cigarettes have often been used by smokers as a harm-reduction intervention aimed to assist with cigarette-smoking cessation^5^. A meta-analysis found that e-cigarette users (who received free e-cigarettes in trials) were 1.5 times more likely to quit smoking than the control group^6^. Thus, they may play a role in smoking cessation in adult smokers and the benefits of use may outweigh the risks from a public health-harm reduction perspective as they are a safer alternative^7^. However, e-cigarettes are increasingly initiated by teenagers, some of whom have never previously smoked^8^ and who are exposed to unnecessary health risks associated with e-cigarette use, making them a public health issue^9^.

**Table 1:** Common electronic cigarette related terms^2–4,37,124,125^.

Term	Meaning
Electronic cigarettes	A battery operated device, which heats a liquid, commonly containing glycerol, glycol, nicotine, flavorings, and other additives, producing an aerosol, which the user inhales with the mouthpiece. The cartridge is either refillable or disposable.
E-cigarettes/E-cigs	Short terminology for electronic cigarettes
Vape pens	Another term for e-cigarettes that are shaped like or resemble pens
Pods	Another term for e-cigarettes
Vape products	A term which broadly includes all forms of electronic cigarettes made by different manufacturers including nicotine free e- cigarettes and ones with nicotine along with other chemical additives
Vaping	The act of (verb) using electronic cigarettes/ vape products by inhalation of the smoke free aerosol that is generated from the device
Juice	The liquid that is added to electronic cigarettes, which is heated for inhalation by the user. It can include nicotine, cannabis oil, flavorings, and other chemical additives.
JUUL	A discrete looking electronic cigarette device which resembles a USB drive and has a high content of nicotine
EVALI	Electronic Cigarette of Vaping Related Lung Injury resulting from the inhalation of vitamin E acetate and cannabis using electronic cigarettes

Some of the reported reasons for e-cigarette use in teenagers and young adults include their flavoring^10,11^, discreteness^12^, easy accessibility^10^, desire to experiment^10^, perceptions that they are safer^10^, and advertising as well as marketing that directly targets young people^13^. Research on flavoring found that sweet flavors (e.g., fruity or candy flavored) were more often selected by teenagers over tobacco or minty flavored (conventional) e-cigarettes^14^.

Here, we review of the epidemiology of e-cigarette use in teenagers and young adults and associated health risks, theoretical mechanisms, and management, including prevention as well as interventions and policies. The overarching aim is to provide an in-depth overview of e-cigarette usage in teenagers and young adults from a public health perspective and to provide insight into emerging trends as well as opportunities for health promotion.

## Methods

A review of PubMed (Medline) and Google Scholar was undertaken in September 2021. We broadly included all up-to-date studies that were related to teenage-vaping epidemiology, mechanisms, and global policies published in the English language. Primary studies that were not undertaken in teenager ages 13–18 or young-adult ages 19–24 were excluded. Systematic reviews and meta-analyses were only included if they were related to global policies or epidemiological updated findings related to our study population or highly applicable to it. Studies on youth perceptions of e-cigarettes were only included if the papers addressed policy.

We used broad search terms that included word variations for “e-cigarettes” or “vaping”, “teenagers”, “respiratory health effects”, and “vaping policies”. MESH terminology and free text was used in the search. A medical librarian assisted with the search strategy. Manual hand and primary government-database searches were also undertaken. The details of the Medline search-strategy example are summarized in Table 2.

**Table 2:** PubMed/Medline search strategy.

Topic	Keywords
Population	“adolescent”[MeSH Terms] OR “adolescen*”[All Fields] OR “youth”[tiab] OR “youths”[tiab] OR “teen”[tiab] OR pubescen*[tiab] OR puberty[tiab] OR minor[tiab] OR minors[tiab] OR underage*[tiab] OR “under age*”[tiab] OR “young adult”[mesh] OR young adult*[tiab] OR “high school*”[tiab] OR student*[tiab] AND
Intervention	(“Counseling”[Mesh] OR counsel*[tiab]) OR (((reduc*[ti] OR decreas*[ti]) AND (use[tiab] OR utilization[tiab]))) OR (“harm reduction*”[tiab] OR “Mass Screening”[Mesh] OR “screen*”[tiab] OR “routine testing”[tiab] OR experiment*[tiab] OR intervention*[tiab] OR study[tiab] OR studies[tiab] OR trial*[tiab] OR RCT[tiab] OR random*[tiab] OR “secondary prevention”[mesh] OR “tertiary prevention”[mesh] OR quit*[tiab] OR stop[ti] OR stopped[ti] OR stopping[ti] OR stops[ti]) OR (“Health campaign*”[tiab] OR “education campaign*”[tiab] OR “educational campaign*”[tiab] OR “media campaign*”[tiab]) OR (((Public health[ti] OR regulat*[ti]) AND (effect*[tiab] OR impact*[tiab] OR strateg*[tiab] OR campaign*[tiab] OR policy[tiab] OR policies[tiab] OR program*[tiab]))) OR (Taper*[tiab] OR “Behavioral support*”[tiab] OR “behavioral support*”[tiab] OR “Behavioral therap*”[tiab] OR “Behavioral therap*”[tiab] OR manag*[ti] OR control*[ti]) OR (((“flavoring agents”[mesh] OR flavor*[tiab] OR flavor*[tiab]) AND (restrict*[tiab] OR limit*[tiab] OR decreas*[tiab]))) OR (“reduced risk”[tiab] OR “risk reduction”[tiab] OR “vaping cessation”[tiab] OR “cessation therap*” OR “cessation treatment*”[tiab]) OR ((increas*[tiab] AND price[tiab])) OR (“smoke free law”[tiab] OR “smoke free laws”[tiab] OR “population health standard*”[tiab] OR “warning label*”[tiab] OR tax[tiab] OR taxation[tiab] OR taxed[tiab] OR taxes[tiab] OR taxing[tiab] OR tax[tiab] OR taxation[tiab] OR taxed[tiab] OR taxes[tiab] OR taxing[tiab] OR “Commerce”[Mesh]) OR (((“Flavoring Agents”[Mesh] OR flavor*[tiab] OR flavor*[tiab]) AND (restricted[tiab] OR restriction[tiab] OR limit*[tiab] OR decreas*[tiab]) OR (increas*[tiab] AND price[tiab]))) OR (((reduc*[tiab] OR decreas*[tiab]) AND (use[tiab] OR utilization[tiab]))) OR (“Government Regulation”[Mesh] OR “Public Policy”[Mesh] OR “legislation and jurisprudence”[MeSH Subheading]) OR (“prevention and control”[sh] OR “public health”[mesh:noexp] OR “consumer product safety”[mesh] OR “public health practice”[mesh]) AND
Outcome	“adverse effects” OR “Lung injury” OR “EVALI” OR “toxicity”[Subheading] OR “poisoning” OR “irritation” OR “inflammation” OR “pneumonia” OR “allergy” OR intoxication OR “respiratory effect” OR “cardiovascular” OR “health effect*” OR “case report*” OR “adverse effects”[Subheading] OR “lung injury”[MeSH Terms] OR “poisoning”[MeSH Terms] OR “poisoning”[Subheading] OR “poisons”[MeSH Terms] OR “poisons”[All Fields] OR “irritants”[MeSH Terms] OR “inflammation”[MeSH Terms] OR “pneumonia”[MeSH Terms] OR “hypersensitivity”[MeSH Terms] OR “allergy and immunology”[MeSH Terms] OR “cardiovascular system”[MeSH Terms] OR casereports[Filter] Combined into one large search string

**Fig. 1:**
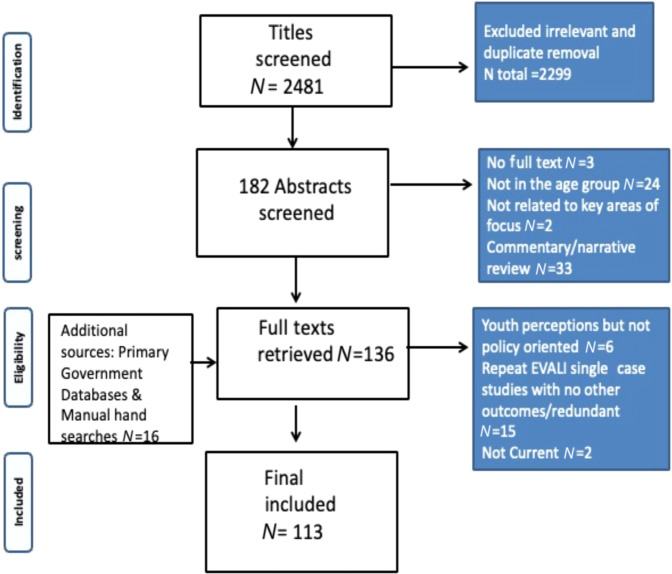
Prisma flow chart.

After screening 2481 titles against the inclusion and exclusion criteria, followed by abstract screening and full-text retrieval, 113 studies were included in the final review. Figure 1 illustrates the search process (PRISMA flow chart)^15^.

### Reporting summary

Further information on research design is available in the Nature Research Reporting Summary linked to this article.

## Results

### Epidemiology

The uptake of e-cigarettes has markedly increased in recent years across the globe^16^. A systematic review found that e-cigarette usage in teenagers increased by over 19% between 2011 and 2018 in the United States^12^. Studies have also reported an increase in the prevalence of use in Canada and the United Kingdom^9^. According to the report from the Canadian Student Tobacco, Alcohol, and Drugs Survey, the prevalence of e-cigarette usage in teens (grades 7–12) within the past 30 days was 20 percent in 2018–2019, a doubling of the prevalence in the previous year’s report^17^. A total of 90% had consumed products containing nicotine^17^. Between 2010 and 2014, there was a 24.4% rise in e-cigarette use among teens in Eastern and Central Europe^18^. The study had also found that a large proportion of students (43.7%) had previously tried e-cigarettes^18^, highlighting that many young adults have previously experimented with e-cigarettes.

Prevalence of ever use of e-cigarettes appears to be lower in Asian countries such as Japan, where 3.5% reported past use, and South Korea, where 10.1% reported previous experimentation with e-cigarettes^19,20^. One study in China reported a low prevalence of past 30-day use of 1.2%, though the study was undertaken in middle-school students instead of high-school students, which could have underestimated teenage e-cigarette use^21^. However, more studies are needed in this region to better ascertain the prevalence of use and changes over time. Data from South America are further limited, but older studies in Brazil (2015) indicate that 2.1% had ever tried e-cigarettes^22^. Additionally, there was a reported rise in prevalence of teenage e-cigarette usage in Argentina between 2014 and 2015 of 5.2%^23^. There are fewer studies in low-income countries, in particular in Africa and India^24^, where the prevalence of e-cigarette use in teenagers is underreported. There is a gap in the literature in low-income countries, highlighting that the topic of e-cigarette use in teenagers remains relatively unexplored and more research is needed in this area.

**Fig. 2:**
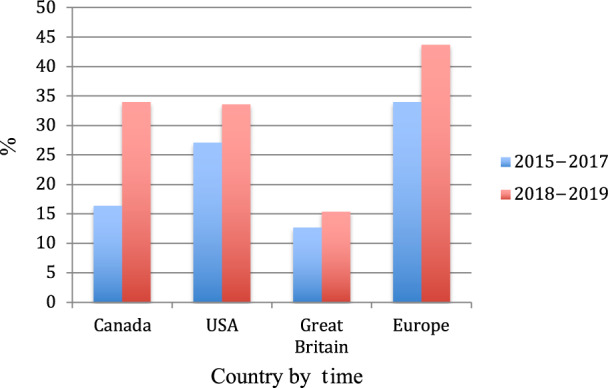
History of “Ever Use” of e-cigarettes in teenagers by country and period^9,18,25–27,30,33^. History of “ever use” of e-cigarettes in Canada, the United States, Great Britain, and the rest of Europe between 2015–2017 and 2018–2019^9,18,25–27,30,33^. If countries reported history of past use within any of these time periods, they were included. Please note that the 2015–2017 prevalence of ever use is for the following European countries: Belgium, Finland, Germany, Ireland, Italy, the Netherlands, and Portugal^28^. The 2018–2019 report in Europe collected data from Central and Eastern Europe, including the following countries: Poland, Lithuania, Belarus, Slovakia, and Russia^18^. It should be noted that while the report by Hammond et al^9^ reported a prevalence of “ever use” in the United Kingdom of 32.7% (2018), the Action and Smoking on Health Report in England^25^ produced a significantly lower prevalence of 16.4% for the same period in Great Britain. It could be that Northern Ireland has a higher prevalence of ever use and was omitted from the report.

**Fig. 3:**
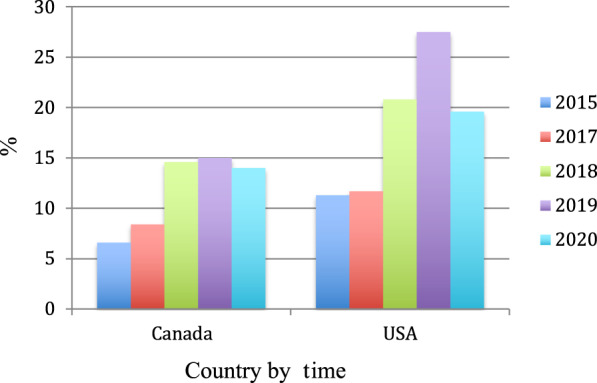
Prevalence of e-cigarette use (past 30 days) in teenagers in North America over a five year period (between 2015 and 2020)^9,27,29–33^. Past 30-day prevalence of e-cigarette use from 2015 to 2020 in North America (comparisons between Canada and the United States) in teenagers (grades 7 through 12). Reported prevalence declined in 2020 during the COVID-19 pandemic. The Canadian Tobacco and Nicotine Surveys were used for 2015, 2019, and 2020 surveys, respectively^27,29^. The 2018–2019 surveys for Canada were obtained from the International Tobacco Control Policy Evaluation Project (ITC) Youth Tobacco and Vaping Survey, in Hammond et al^9^. The NYTS^30^ (in the Surgeon’s Report on E-Cigarette Usage), CDC, and FDA data from reports^31,32^ between 2015 and 2020 were also used. It should be noted that there is a slight discrepancy in reported past 30-day prevalence of use in Canada between a Canadian report in 2017^33^ and the ITC survey report^9^ (6.6% versus 8.4%) as well as between the FDA USA^32^ report and the ITC in 2019^9^ (20.8 versus 16.2%).

Figure 2 compares reported proportions of “ever use” of e-cigarettes in teenagers across high-income countries, including Canada, the United States, Great Britain, and Europe between 2015–2017 and 2018–2019^25–28^. Figure 3. compares trends in past 30-day prevalence of e-cigarettes from 2015 to 2020 in North America. Overall, the trends indicate a rise in prevalence and past use of e-cigarettes across countries^9,27,29–33^, though prevalence of use declined in 2020 during the pandemic according to data from Canada and the United States^27,31^.

#### Health effects and associated risks

Although e-cigarettes appear to be a safer alternative than smoking cigarettes over the short term^7^, they are not without risks, especially when used on a regular basis^34^. The potential benefits and risks of e-cigarettes are summarized in Table 3. Previous reviews have linked e-cigarettes with asthma and chronic obstructive pulmonary disease^34^. A systematic review found that e-cigarettes were associated with myriad respiratory health effects such as exacerbations of asthma, eosinophilic pneumonia, epiglottitis, bronchitis, and acute respiratory distress^35^. Other notable symptoms in regular teenage vapers have included headaches, generalized coughing, insomnia, weakness, and pain in the chest area^36^.

**Table 3:** E-Cigarettes Potential Harms and Benefits^6,7,35,37,46–48,51,62,63,72^.

Exposure	Potential benefits	Potential risks
E-Cigarettes	• May assist with smoking cessation • Potential harm reduction intervention for cigarette smokers as a form of nicotine replacement (also mimics smoking hand to mouth behaviors) • Safer alternative than smoking cigarettes • Less toxic chemicals and in lower doses than in conventional cigarettes	• Exposure of e-cigarettes to minors (children and teenagers) and previous nonsmokers • Potential gateway to smoking and initiation of other substances in teenagers • Dual smoking and e-cigarette use compounds public health risks • May increase the risk of respiratory health problems • May increase the risk deficits in cognition, brain development, effort-reward imbalances in the brain in children and teenagers • Long-term effects on health are unknown

The FDA had issued a warning in 2019, after a series of cases (N = > 1000 of E-cigarette and vaping use associated lung injury (EVALI))^37^, which were later confirmed to have been caused by the addition of THC and vitamin-E acetate to vape products^38–40^. The specific effects of e-cigarettes on lung injury in teenagers (seven case series) included tachycardia, shortness of breath, and coughing^41^. Six out of the seven cases required ventilator support and were hospitalized^41^.The odds of getting COVID were also five times greater in teenage vapers relative to their nonvaping counterparts (OR = 5.0; 95% CI = 1.8–14.0)^42^. A total of 25.8% of participants who reported previous vaping had symptoms of COVID when compared with nonvapers (13.5%)^42^. It should be noted, however, that the long-term effects of e-cigarettes on respiratory health cannot yet be ascertained^43^.

There is some emerging research, which suggests that e-cigarettes may have cardiovascular effects in teenagers. A study found a rise in arterial blood pressure and heart rate in young adult vapers using JUUL, but not in e-cigarettes without nicotine^44^. Cardiopulmonary risk is also compounded in dual e-cigarette and cigarette smokers^45^.

In addition to this, nicotine use has been documented to have adverse effects on cognition and the developing adolescent brain^46–48^, as well as fetal brain development^46^. Research in teenagers suggests that it is associated with memory problems and troubles with concentrating and focusing on tasks, with increased impulsive behaviors as adults^48,49^. A review also found that nicotine use was associated with imbalances in brain development, whereby teens exposed to nicotine had less-developed regions in the prefrontal cortex responsible for inhibitory control, while the part of the brain responsible for the reward system (dopamine pathway)^50^ had been well matured as indicated on functional MRIs, highlighting the imbalance in reward and control regions in the brain^47^. Nicotine use during adolescence has also been linked with an increased risk of mental health problems later in life^47,48^.

Furthermore, e-cigarette use is a risk factor for subsequent cigarette smoking. A systematic review and meta-analysis found that e-cigarette users had a 30% chance of initiating cigarette smoking when compared with never-users (7.9%)^51^. The odds of smoking were 3.5-times higher (95% CI = 2.4–5.2) in e-cigarette users when compared with never-users (23.2% of previous e-cigarette users reported smoking versus 7.2% of never-users)^51^. Research in young adults found that 82.6% of e-cigarette consumers concurrently used additional nicotine products such as conventional cigarettes. Prevalence of nicotine dependence in this young population was 68%^52^. Another study found that nearly half of teenage vapers smoked a cigarette two years later when compared with their nonvaping counterparts^53^. A qualitative study in teens found that many identified e-cigarettes as a gateway to cigarette smoking^54^.

E-cigarettes are also associated with downstream substance use. Research has found that teenagers who use e-cigarettes are also more likely to use cannabis when compared with non-e-cigarette users and that it is commonly added to vaping products^52,55^. Cannabis vaping has been linked with bronchitis in youth as well^56^.

**Fig. 4:**
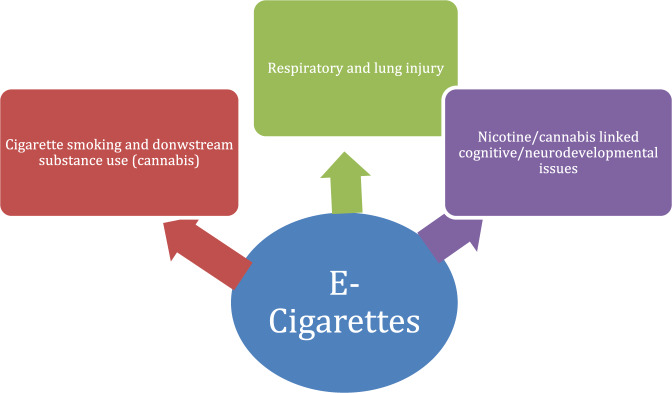
The health risks associated with vaping^37,46–48,51,52,55^. The following figure illustrates the relationship between e-cigarette exposure and potential health effects in teenagers, which primarily affect the respiratory system, neurodevelopment/cognition, and may increase the risk of dual smoking and addiction to other drugs.

Finally, there have been incidents of ingestion and intoxication associated with e-cigarettes in preteens^57^. Figure 4 illustrates the health risks associated with vaping.

#### Mechanisms

Nicotine is a well-established respiratory irritant^58^, but other chemicals in e-cigarettes (e.g., diacetyl^59^, propylene glycol, carbon monoxide, and formaldehyde^60^) also have detrimental effects for lung function including respiratory volume^61^. E-cigarettes also contain trace amounts of toxic chemicals such as polycyclic aromatic compounds in tandem with heavy metals, aldehydes, and nicotine derivatives^62^. However, it should be noted that exposure to potentially toxic chemicals is lower in e-cigarettes than in conventional cigarettes^63^. E-cigarettes also irritate mucous membranes and trigger the release of inflammatory markers^64^. Additionally, the sweet-flavoring additives (e.g., candy or fruity flavored) have also been reported to be hazardous to the lung^65^. The cinnamon-flavoring cinnamaldehyde has been identified as being one of the main constituents capable of damaging immune cells in the lungs (macrophage-phagocytosis impairment) even without nicotine as a co-additive^65^. Furthermore, the sweet Crème Brulee flavoring was linked with increased tumor-necrosis factor, interleukin levels, and oxidative stress associated with DNA changes^66^. In terms of e-cigarette or vaping use-associated lung injury (EVALI), vitamin E acetate along with cannabis oil were identified as being the primary causative agents^38,39^.

In addition to this, nicotine is a risk factor for cardiovascular disease through its well-known effects on endothelial function and stimulation of inflammatory markers such as C-reactive protein^67,68^. Studies in youth have identified a possible mechanism for cardiovascular effects resulting from activation of the splenocardiac axis from inhaled toxins in e-cigarettes^69^.

Nicotine additionally affects the developing brain through its effect on cerebral cortex as well as in the hippocampus^49^. All types of e-cigarettes, including non-nicotine ones, have been reported to induce oxidative stress, thereby increasing the risk of cognitive-related impairment in teenagers^70^. Research also suggests that nicotine can bind to N-acetylcholine receptors, thereby impacting signaling in the prefrontal cortex^47^. Nicotine also has been documented to have an effect on serotonin receptors (5HT1 and 5HT2), which subsequently affects the body’s response to serotonin, supporting the link between exposure to nicotine in adolescence and risk of mood disorders later in life^47,48^.

Furthermore, e-cigarettes are thought to increase dual smoking and downstream substance use through the gateway hypothesis, whereby exposure to nicotine products further puts individuals at risk of initiating other substances by stimulating neurotransmitters associated with the reward system^4,55,71–73^. This feedback loop creates a pathway for substance abuse and dependence^72^.

There is some evidence of second-hand exposure effects, but the exposure dose is much smaller than in conventional cigarettes^74^. However, a study found that teenagers presenting with an asthma attack over a 12-month period were 27% times more likely to be exposed to e-cigarette second-hand smoke relative to their counterparts^75^. Thus, second-hand exposure may be related to respiratory health in youth, including asthma and generalized wheezing^75,76^.

### Screening, prevention, and management

Research indicates that screening patients for e-cigarette usage in primary practice is not frequently undertaken by medical practitioners^77^. One study found a low prevalence of screening for e-cigarettes in primary-care practice relative to smoking screening (14% versus 86%) in a sample of 776 practitioners across the United States^77^. This low uptake is concerning, given the serious health risks of e-cigarettes. A qualitative study in the United States further confirmed that there is insufficient knowledge of e-cigarettes among physicians, including both the potential benefits and health risks^78^. A study in US college students found that most students did not receive any form of counseling about risks from medical practitioners, including dental hygienists^79^. More research is needed to learn about the global screening prevalence of e-cigarette use in primary care. Studies have also shown that there is a need for stronger education on e-cigarettes in medical curricula, which will allow physicians to begin addressing e-cigarette use in teenagers^80^.

Presently, there is little information on primary-care interventions for e-cigarette use in teenagers and young adults. A case study of a 23-year-old e-cigarette user shows promising results for tapering e-cigarette use with the assistance of a pharmacist^81^, which suggests that different healthcare practitioners may play a role helping patients with gradually tapering off e-cigarettes. A randomized controlled trial of asthmatic teenagers who attended one of four clinics found that physicians discussed smoking during 38.2% of thee visits, but vaping was never brought up as a topic^82^. This emphasizes that physicians should discuss both smoking and vaping during appointments^82^, in particular in youth presenting with asthma^75^.

Medical curricula should stress that concurrent smoking and vaping screening and management interventions should be undertaken in the primary-care setting. This way, many cases will not be missed given the high prevalence of dual use^51^. Family physicians should aim to identify youth at risk of vaping through screening questionnaires and aim to increase awareness of vaping for prevention purposes. This could include handing out brochures to patients and their families about the health risks associated with vaping and therapies that are available, which can assist with gradual tapering of nicotine from e-cigarettes. Family physicians have previously recommended open discussions with youth about risks during appointments^83^, as well as educating families through public health educational campaigns^84^.

It may also be strategic for medical, public health practitioners, and researchers to target particular groups and populations of teenagers that are most vulnerable to using e-cigarettes. A longitudinal study in the United Kingdom found an association between socioeconomic disadvantage and e-cigarette use in teenagers and young adults^85^. A systematic review also found that older teenagers from more affluent homes, of white ethnicity, and with higher levels of education had higher levels of knowledge and awareness of e-cigarette use, highlighting a possible need to educate younger teenagers with less education, ethnic minorities, and from lower-income neighborhoods^86^. It should be noted that one study found conflicting results with regard to the relationship between SES and e-cigarettes, whereby young adults from wealthier families were more likely to use e-cigarette, though the comparison groups were all in the affluent state of Connecticut^87^.

Education was also found to be inversely associated with e-cigarette use in another study, but it had the greatest association in whites when compared with black young adults^88^. Vocational training, without higher education, was found to also be associated with e-cigarette use in youth in Europe^89^. Thus, public health campaigns and medical doctors could potentially target individuals with lower levels of education, lower SES, and racially diverse groups to minimize any potential inequities in health.

Gender differences in e-cigarette use have also been noted in North America as well as Europe, whereby males were more likely to use them^89–91^. Additionally, since research indicates that females use e-cigarettes for mostly weight and stress management^92^, interventions could focus on assisting them with stress along with making healthy lifestyle choices associated with weight.

Other particularly vulnerable groups have also included teenagers with impulsivity as well as those with mental health problems^93–95^. A study that explored EVALI cases found that mental health problems were prevalent in this population^95^. Thus, physicians and public health researchers may also consider screening and targeting individuals with mental health problems.

To date, there have been limited community-based and public health intervention trials to assist with e-cigarette prevention. “Catch my breath” was a prevention intervention in 12 middle schools across the United States. The intervention focused on increasing knowledge on the harms associated with e-cigarette use^96^. The study authors found statistically significant differences in e-cigarette use prevalence in schools that had implemented the program when compared with control schools. They also found increased knowledge of e-cigarettes and the risks associated with their use^96^.

Similarly, public health interventions targeting existing teenage users are in their infancy. There is a current text messaging intervention for e-cigarette cessation in teens in the United States^97^. The intervention provides users with educational content on e-cigarettes, focuses on fostering self-efficacy, assists with resilience building, and provides users with support and encouragement. The study had a very high enrollment after about one month of recruitment, with over 27,000 teenagers and young adults enrolled^97^. This indicates that this form of intervention is feasible, given the willingness for e-cigarette users to enroll^97^. Previous studies have found that text messaging for smoking cessation is effective and acceptable for this population^98–101^, indicating that it could be used for vaping.

Additionally, there are very few commercially available e-cigarette cessation apps that can help teenagers and young adults quit. A systematic review of apps in the Google Play Store found that most apps encouraged e-cigarette use and that only 2 out of 79 were vaping cessation apps^102^. There is a need to develop an app that can be readily available and accessible to teenagers wanting to quit as well as an educational prevention app.

### Policies

Strict policies to limit e-cigarette accessibility and exposure play an important role in preventing use. Research indicates that children and teenagers are exposed to e-cigarette marketing^103^. A study in the United Kingdom found that most e-cigarette advertisements were near children’s stores and in areas that were less affluent^103^, indicating that social health inequalities may exist, but more research is needed in this area. A review of 124 e-cigarette marketing publications revealed that companies have increased expenditures on social media campaigns and that they are often marketed as an alternative to cigarette smoking^104^. This is especially concerning given how social media may influence the decisions of teenagers and young adults. A randomized controlled trial found that by exposing youth without prior smoking history (*N* = 417) to e-cigarette advertising (four advertisements), they were more likely to select e-cigarettes and have positive attitudes toward them relative to controls not exposed to this advertising^13^. Research had found that many e-cigarette advertisements on social media had used cartoons on packages to promote vaping in youth along with hashtags for vaping (#ejuice and #eliquid^)^^105^. The study authors also found that over 20% of advertisements had used a cartoon (66% of which were promotional posts), indicating that youth are often the targets of these ads across the globally accessible Instagram platform. They recommend similar policies to the ones for smoking including the Historical Master Settlement Agreement that banned advertising to youth^105^. Studies have also found that teenagers require multiple warnings in the forms of messages and ads to reduce their positive interest and susceptibility to e-cigarettes^106^ and that perceptions of safety are related to environmental policy restrictions on vaping^107^.

Research also indicates that patterns of e-cigarette use changed markedly in teenagers and young adults during the COVID-19 pandemic^108^. Changes in substance use behavioral patterns included ordering from alternative sellers, buying vaping products online, quitting vaping, and switching to cannabis or other products, resulting from the inherent challenges with making purchases at local vendors^108^. This emphasizes how the availability of vaping products including their placement and immediate accessibility influences e-cigarette behavioral patterns, including quitting^108^.

Besides restricting marketing and advertisements, limiting the availability of e-cigarettes and accessibility to teenagers is greatly needed. A policy review on bans on the sale of e-cigarettes to minors across the United States found that e-cigarette use decreased along with smoking traditional cigarettes^109^. A qualitative study of adult vapers found that many agree with bans on advertising to minors to protect them^110^.

A review of global vaping policies found that 68 countries regulate e-cigarettes and that the most frequent cross-national governmental policies include age limits (over 18 years of age), restricting advertisements, and placing bans on vaping in public places, while e-cigarette taxes are not commonly used^111^. The review found that Australia, the Czech Republic, and Malaysia classified e-cigarettes as toxic and poisonous substances^111^. Countries that have enacted child safety policies to protect children include Canada (banned flavoring and marketing to children)^112^, Australia (available by prescription only with a child safety seal)^113^, New Zealand (banned vaping near schools)^114^, the United Kingdom^111^, the United States (some states have banned JUUL)^115^, Finland, Germany, Ireland, Italy, Lithuania, Malta, Netherlands, and the Philippines^111^. Some countries with vape-free restrictions that were also identified include France, Germany, Greece, Jamaica, Nepal, Portugal, Slovakia, Spain, Turkey, Venezuela, and Vietnam^111^. Countries with taxes on e-cigarettes include Italy, Latvia, Portugal, Republic of Korea, Togo, and the United Kingdom^111^. Asian countries that have banned e-cigarettes include Singapore and Thailand, and Japan has banned the use of nicotine-containing e-cigarettes but not e-cigarettes without nicotine^116^. Vaping products are also prohibited in the United Arab Emirates^116^. Switzerland had banned the sale of vaping products until 2018, but now they are available on the market^117^.

In developing countries, where resources are depleted and there is less regulatory oversight^118^, concerns are raised about efforts to protect minors. Although data in India are limited, protective measures have nonetheless have been put into place in 2019, when e-cigarettes were banned to protect minors^119^. Concern has been raised in Guatemala over the lack of regulatory control over flavored e-cigarettes that are enticing for teenagers^120^. While little is known about Africa, South Africa is planning on placing restrictions on e-cigarettes in 2021^121^, but there has been strong opposition from the Tobacco Industry^122^.

## Discussion

### Recommendations

Without stricter interventions and policies, teenagers and young adult vapers will continue to be at risk of multiple health problems associated with e-cigarettes.

The following are a set of recommendations:

1. *Strengthen global policies to restrict marketing, use of enticing flavoring, accessibility, and exposure to e-cigarettes in the environment*
2. *Increase physician education on screening and nicotine tapering in the primary-care setting*.
3. *Increase public health education campaigns and develop evidence-based interventions*.
4. *Develop collaborations between physicians and public health researchers through joint efforts in education, screening, and referral*.

**Fig. 5:**
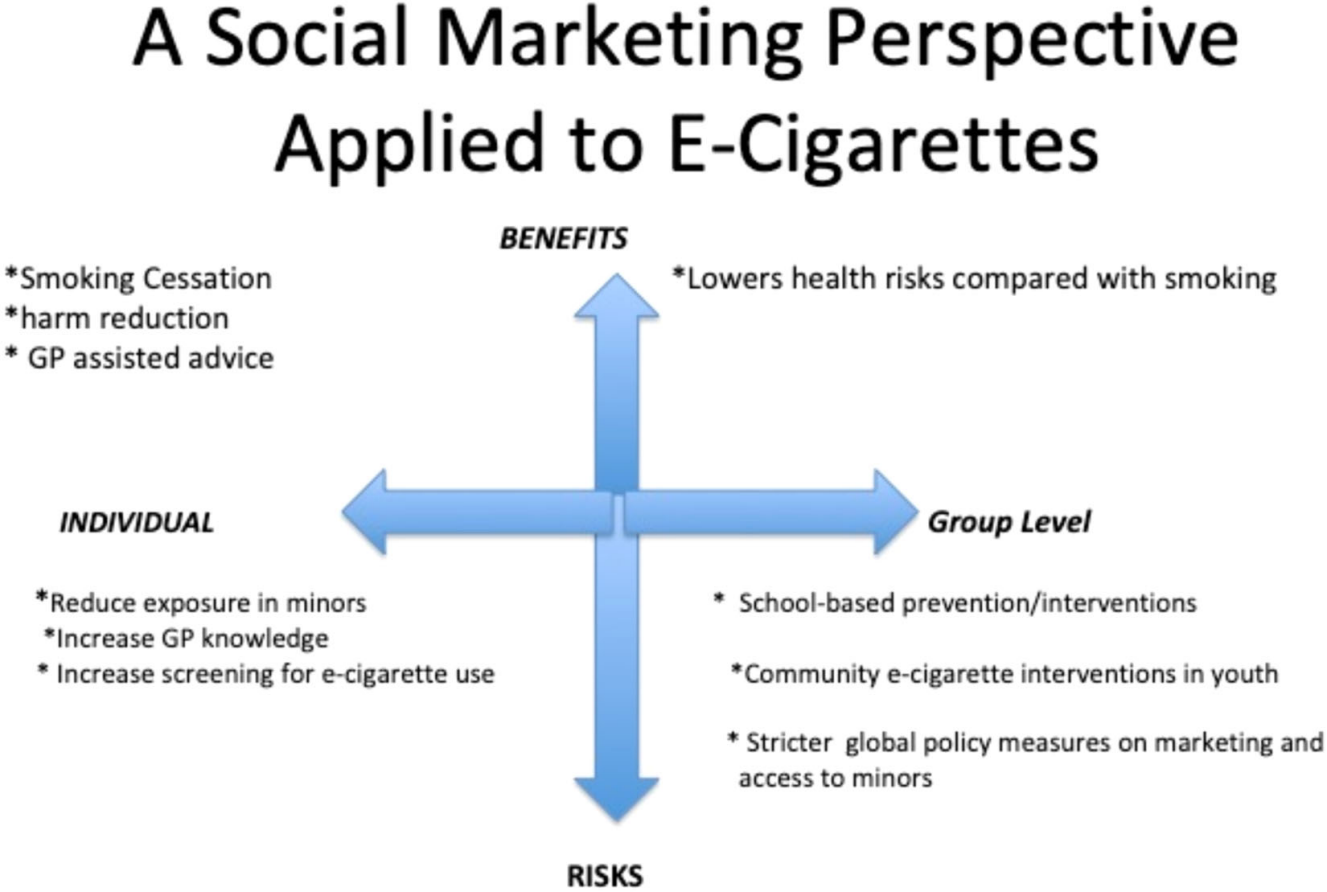
Strategies to reduce the harm and maximize the benefits associated with e-cigarettes at the individual and group levels^82,96,97,123^. The North Axis represents the benefits of e-cigarettes for smokers and the South axis represents the risks, while the East and West axes represent the strategies that may be adopted at an individual level and community/population level. By maximizing the benefits in select adult smokers through harm reduction and minimizing the risks of exposure in minors, e-cigarettes may be safely used.

**Fig. 6:**
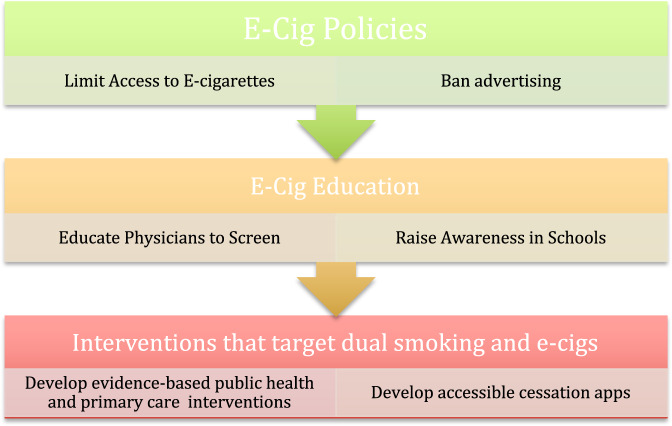
A three-tiered approach to managing e-cigarette use in minors^126^. The following figure illustrates a three-tiered approach to managing e-cigarette use in minors, which includes policy changes, awareness, and prevention campaigns, and finally public health interventions that target existing teenage users.

Figure 5 illustrates strategies that may be applied from a social-marketing perspective^123^ to e-cigarettes by emphasizing that the health risks^41,46,51^ should be reduced by restricting their access to children and teenagers^9^, while the benefits of their use may be maximized when safely used in adult smokers attempting to quit^7^. It illustrates that screening, prevention, and intervention can take place in primary-care settings and through public health interventions. Figure 6 illustrates a three-tiered approach to screening, education, prevention, and interventions for e-cigarettes in youth.

## Conclusion

In summary, e-cigarettes pose a health threat to teenagers and young adults, given the rise in the prevalence of use. While e-cigarettes are a safer alternative than smoking cigarettes and may be used as a harm-reduction strategy in existing smokers, measures need to be urgently put into place to protect children and teenagers from unnecessary use and potential dual smoking and e-cigarette uptake. The outlook depends on whether sufficient primary care and public health strategies will be implemented to protect minors and young adults. As the long-term effects are unknown^62^, it is especially prudent to limit unnecessary exposure. There is an urgent need to develop evidence-based primary-care intervention and public health interventions that target vulnerable groups. Furthermore, there is need for stronger public health protection policies and bans to protect youth.

## Supplementary information

original submission cover letter copy

original submission manuscript copy

Checklist for NPJRM

NPJRM Editorial Policy Checklist

NPRJM Reporting summary checklist

## Data Availability

No datasets were generated nor analyzed from this study. Source data for Figs. 2–3 are detailed in the paper (i.e., data on vaping prevalence are available on the CDC and FDA websites).
