# Supplementary material for: Global youth vaping and respiratory health: epidemiology, interventions, and policies
Source: NPJ Prim Care Respir Med. 2022 Apr 11;32:14. doi: 10.1038/s41533-022-00277-9 (PMC9001701; doi:10.1038/s41533-022-00277-9)
Supplement: Supplementary file 2 — original submission manuscript copy [file 41533_2022_277_MOESM2_ESM.pdf]

Teenage and Young Adult Vaping and Respiratory Health: ***An Updated Review of Global Epidemiological Trends, Interventions, and Policy Approaches***

Lynnette Nathalie Lyzwinski PhD MPhil PH[1], John Naslund PhD[2], Christopher J Miller PhD[3], Mark J. Eisenberg MD MPH[4]

[1]Center for Clinical Epidemiology, Lady Davis Institute, Jewish General Hospital, Montreal, Quebec, Canada and McGill Medical School, McGill University

[2]Global Health and Social Medicine (Blavatnik Institute), Harvard Medical School, Harvard University, Cambridge Massachusetts, USA

[3]Department of Psychiatry, Harvard Medical School, Harvard University, Cambridge Massachusetts

[4]Division of Cardiology, Jewish General Hospital, McGill University, Montreal, Quebec, Canada Departments of Medicine and of Epidemiology, Biostatistics and Occupational Health, McGill University, Montreal, Quebec, Canada

Address for Correspondence:

Lynnette Lyzwinski, PhD, MPhil PH
Centre for Clinical Epidemiology
McGill University
School of Medicine
Telephone: (514) 340-8222 Ext.23564
E-mail: Lynnette.Lyzwinski@mail.mcgill.ca

Abstract

E-cigarette usage (also known as e-cigarettes or vaping products) has increasingly been recognized as a global public health problem. One challenge in particular involves their marketing to minors (teenagers and children) and the rising prevalence of use in this population. E-cigarettes unnecessarily expose minors to health risks; these include respiratory health problems, such as exacerbations of asthma, bronchitis, and respiratory tract irritation. Nicotine, commonly found in e-cigarettes, is also associated with cognitive impairment and neurodevelopmental problems. E-cigarettes are also risk factors for downstream substance use including cigarettes and cannabis initiation (the gateway hypothesis), which compounds health risks in dual users. Current public health preventative and intervention studies are limited, and there is a clear need for more interventions that may prevent usage and assist with cessation in this vulnerable population. Physician education and screening uptake should also be enhanced. Stricter public health policy and protection measures are also needed on a global scale to limit e-cigarette exposure in minors.

INTRODUCTION

The use of electronic cigarettes (also known as e-cigarettes, e-cigs, or vaping products) has increasingly been recognized as a global public health problem[1]. Vaping consists of inhaling a smoke free aerosol through a mouthpiece, which is produced through the heating of a liquid such as glycol or glycerine in an electronic device[2, 3]. Most e-cigarettes have the shape of a pen, but others are more discrete looking such as JUUL, which resembles a USB drive and is popular among teenagers[4]. Common terminology for e-cigarettes is summarized in Table 1. E-cigarettes have often been used by smokers as a harm reduction intervention aimed to assist with cigarette smoking cessation[5]. A meta-analysis found that e-cigarette users (who received free e-cigarettes in trials) were 1.5 times more likely to quit smoking than the control group[6]. Thus, they may play a role in smoking cessation in adult smokers and the benefits of use may outweigh the risks from a public health harm reduction perspective as they are a safer alternative[7]. However, e-cigarettes are increasingly initiated by teenagers, some of whom have never previously smoked[8] and who are exposed to unnecessary health risks associated with e-cigarette use, making them a public health issue[9].

Some of the reported reasons for e-cigarette use in teenagers and young adults include their flavouring[10, 11], discreteness[12], easy accessibility[10], desire to experiment[10], perceptions that they are safer[10], and advertising as well as marketing that directly targets young people[13] Research on flavouring found that sweet flavours (e.g., fruity or candy flavoured) were more often selected by teenagers over tobacco or minty flavoured (conventional) e-cigarettes[14].

Here, we review of the epidemiology of e-cigarette use in teenagers and young adults and associated health risks, theoretical mechanisms, and management, including prevention as well as interventions and policies. The overarching aim is to provide an in-depth overview of e-cigarette usage in teenagers and young adults from a public health perspective and to provide insight into emerging trends as well as opportunities for health promotion.

METHODS

A review of PubMed (Medline) and Google Scholar was undertaken in September 2021. We broadly included all up-to-date studies that were related to teenage vaping epidemiology, mechanisms, and global policies published in the English language. Primary studies that were not undertaken in teenagers ages 13-18 or young adults ages 19-24 were excluded. Systematic reviews and meta-analyses were only included if they were related to global policies or epidemiological updated findings related to our study population or highly applicable to it. Studies on youth perceptions of e-cigarettes were only included if the papers addressed policy.

We used broad search terms which included word variations for “e-cigarettes” or “vaping”, “teenagers”, “respiratory health effects”, and “vaping policies”. MESH terminology and free text was used in the search. A medical librarian assisted with the search strategy.

Manual hand and primary government database searches were also undertaken. The details of the Medline search strategy example are summarized in Table 2.

After screening 2481 titles against the inclusion and exclusion criteria, followed by abstract screening and full text retrieval, 113 studies were included in the final review. Figure 1. Illustrates the search process (PRISMA flow chart).

RESULTS

EPIDEMIOLOGY

The uptake of e-cigarettes has markedly increased in recent years across the globe [15]. A systematic review found that e-cigarette usage in teenagers increased by over 19% between 2011-2018 in the United States[12]. Studies have also reported an increase in the prevalence of use in Canada and the UK[9]. According to the report from the Canadian Student Tobacco, Alcohol, and Drugs Survey, the prevalence of e-cigarette usage in teens (grades 7-12) within the past 30 days was 20 percent in 2018-2019, a doubling of the prevalence in the previous year's report [16]. A total of 90% had consumed products containing nicotine[16]. Between 2010-2014, there was a 24.4% rise in e-cigarette use among teens in Eastern and Central Europe[17]. The study had also found that a large proportion of students (43.7%) had previously tried e-cigarettes [17], highlighting that many young adults have previously experimented with e-cigarettes.

Prevalence of ever use of e-cigarettes appears to be lower in Asian countries such as Japan, where 3.5% reported past use, and South Korea, where 10.1% reported previous experimentation with e-cigarettes[18, 19]. One study in China reported a low prevalence of past 30 day use of 1.2%, though the study was undertaken in middle school students instead of high school students, which could have underestimated teenage e-cigarette use[20]. However, more studies are needed in this region to better ascertain the prevalence of use and changes over time. Data from South America are further limited, but older studies in Brazil (2015) indicate that 2.1% had ever tried e-cigarettes[21]. Additionally, there was a reported rise in prevalence of teenage e-cigarette usage in Argentina between 2014-2015 of 5.2%[22]. There are fewer studies in low income countries, in particular in Africa and India[23], where the prevalence of e-cigarette use in teenagers is underreported. There is a gap in the literature in low-income countries, highlighting that the topic of e-cigarette use in teenagers remains relatively unexplored and more research is needed in this area.

Figure 2. compares reported proportions of "ever use" of e-cigarettes in teenagers across high income countries including Canada, the USA, Great Britain, and Europe between 2015-2017 and 2018-2019[24-27]. Figure 3. compares trends in past 30-day prevalence of e-cigarettes from 2015-2020 in North America. Overall, the trends indicate a rise in prevalence and past use of e-cigarettes across countries[9, 26, 28-32], though prevalence of use declined in 2020 during the pandemic according to data from Canada and the United States[26, 30].

Health Effects and Associated Risks

Although e-cigarettes appear to be a safer alternative than smoking cigarettes over the short-term[7], they are not without risks, especially when used on a regular basis[33]. The potential benefits and risks of e-cigarettes are summarized in Table 3. Previous reviews have linked e-cigarettes with asthma and chronic obstructive pulmonary disease[33]. A systematic review found that e-cigarettes were associated with myriad respiratory health effects such as exacerbations of asthma, eosinophilic pneumonia, epiglottitis, bronchitis, and acute respiratory distress[34]. Other notable symptoms in regular teenage vapers have included headaches, generalized coughing, insomnia, weakness, and pain in the chest area[35].

The FDA had issued a warning in 2019, after a series of cases (N=>1000 of E-Cigarette and Vaping Use Associated Lung Injury (EVALI))[36], which were later confirmed to have been caused by the addition of THC and vitamin E acetate to vape products[37-39]. The specific effects of e-cigarettes on lung injury in teenagers (seven case series) included tachycardia, shortness of breath, and coughing[40]. Six out of the seven cases required ventilator support and were hospitalized[40].The odds of getting COVID were also five times greater in teenage vapers relative to their non-vaping counterparts (OR=5.0; 95% CI=1.8-14.0)[41]. A total of 25.8% of participants who reported previous vaping had symptoms of COVID when compared with non-vapers (13.5%)[41]. It should be noted however, that the long-term effects of e-cigarettes on respiratory health cannot yet be ascertained[42].

There is some emerging research which suggests that e-cigarettes may have cardiovascular effects in teenagers. A study found a rise in arterial blood pressure and heart rate in young adult vapers using JUUL, but not in e-cigarettes without nicotine[43]. Cardiopulmonary risk is also compounded in dual e-cigarette and cigarette smokers[44].

In addition to this, nicotine use has been documented to have adverse effects on cognition and the developing adolescent brain[45-47] as well as fetal brain development[45]. Research in teenagers suggests that it is associated with memory problems and troubles with concentrating and focusing on tasks, with increased impulsive behaviours as adults[47, 48]. A review also found that nicotine use was associated with imbalances in brain development, whereby teens exposed to nicotine had less developed regions in the prefrontal cortex responsible for inhibitory control, while the part of the brain responsible for the reward system (dopamine pathway)[49] had been well matured as indicated on functional MRIs, highlighting the imbalance in reward and control regions in the brain[46]. Nicotine use during adolescence has also been linked with an increased risk of mental health problems later in life[46, 47].

Furthermore, e-cigarette use is a risk factor for subsequent cigarette smoking. A systematic review and meta-analysis found that e-cigarette users had a 30% chance of initiating cigarette smoking when compared with never users (7.9%)[50]. The odds of smoking were 3.5 times higher (95% CI=2.4-5.2) in e-cigarette users when compared with never users (23.2% of previous e-cigarette users reported smoking versus 7.2% of never users)[50]. Research in young adults found that 82.6% of e-cigarette consumers concurrently used additional nicotine products such as conventional cigarettes. Prevalence of nicotine

dependence in this young population was 68%[51]. Another study found that nearly half of teenage vapers smoked a cigarette two years later when compared with their non-vaping counterparts[52]. A qualitative study in teens found that many identified e-cigarettes as a gateway to cigarette smoking[53].

E-cigarettes are also associated with downstream substance use. Research has found that teenagers who use e-cigarettes are also more likely to use cannabis when compared with non-e-cigarette users and that it is commonly added to vaping products[51, 54]. Cannabis vaping has been linked with bronchitis in youth as well[55].

Finally, there have been incidents of ingestion and intoxication associated with e-cigarettes in pre-teens[56]. Figure 4. illustrates the health risks associated with vaping.

MECHANISMS

Nicotine is a well-established respiratory irritant[57], but other chemicals in e-cigarettes (e.g. diacetyl[58], propylene glycol, carbon monoxide, formaldehyde[59]) also have detrimental effects for lung function including respiratory volume[60]. E-cigarettes also contain trace amounts of toxic chemicals such as polycyclic aromatic compounds in tandem with heavy metals, aldehydes, and nicotine derivatives[61]. However, it should be noted that exposure to potentially toxic chemicals is lower in e-cigarettes than in conventional cigarettes[62]. E-cigarettes also irritate mucous membranes and trigger the release of inflammatory markers[63]. Additionally, the sweet flavouring additives (e.g., candy or fruity flavoured) have also been reported to be hazardous to the lung[64]. The cinnamon flavouring cinnamaldehyde has been identified as being one of the main constituents capable of damaging immune cells in the lungs (macrophage phagocytosis impairment) even without nicotine as a co-additive[64]. Furthermore, the sweet Crème Brulee flavouring was linked with increased tumour necrosis factor, interleukin levels, and oxidative stress associated with DNA changes[65]. In terms of e-cigarette or vaping use-associated lung injury (EVALI), Vitamin E acetate along with cannabis oil were identified as being the primary causative agents[37, 38].

In addition to this, nicotine is a risk factor for cardiovascular disease through its well-known effects on endothelial function and stimulation of inflammatory markers such as C reactive protein[66, 67]. Studies in youth have identified a possible mechanism for cardiovascular effects resulting from activation of the splenocardiac axis from inhaled toxins in e-cigarettes[68].

Nicotine additionally affects the developing brain through its effect on cerebral cortex as well as in the hippocampus[48]. All types of e-cigarettes, including non-nicotine ones, have been reported to induce oxidative stress, thereby increasing the risk of cognitive related impairment in teenagers[69]. Research also suggests that nicotine can bind to N-acetylcholine receptors, thereby impacting signalling in the prefrontal cortex[46]. Nicotine also has been documented to have an effect on serotonin receptors (5HT1 and 5HT2), which

subsequently affects the body's response to serotonin, supporting the link between exposure to nicotine in adolescence and risk of mood disorders later in life[46, 47].

Furthermore, e-cigarettes are thought to increase dual smoking and downstream substance use through the gateway hypothesis, whereby exposure to nicotine products further puts individuals at risk of initiating other substances by stimulating neurotransmitters associated with the reward system[4, 54, 70-72]. This feedback loop creates a pathway for substance abuse and dependence[71].

There is some evidence of second-hand exposure effects, but the exposure dose is much smaller than in conventional cigarettes[73]. However, a study found that teenagers presenting with an asthma attack over a 12-month period were 27% times more likely to be exposed to e-cigarette second-hand smoke relative to their counterparts [74]. Thus, second-hand exposure may be related to respiratory health in youth including asthma and generalized wheezing[74, 75] .

SCREENING, PREVENTION, AND MANAGEMENT

Research indicates that screening patients for e-cigarette usage in primary practice is not frequently undertaken by medical practitioners[76]. One study found a low prevalence of screening for e-cigarettes in primary care practice relative to smoking screening (14% versus 86%) in a sample of 776 practitioners across the U.S [76]. This low uptake is concerning given the serious health risks of e-cigarettes. A qualitative study in the U.S further confirmed that there is insufficient knowledge of e-cigarettes among physicians, including both the potential benefits and health risks[77]. A study in US college students found that most students did not receive any form of counselling about risks from medical practitioners including dental hygienists[78]. More research is needed to learn about the global screening prevalence of e-cigarette use in primary care. Studies have also shown that there is a need for stronger education on e-cigarettes in medical curricula, which will allow physicians to begin addressing e-cigarette use in teenagers[79].

Presently there is little information on primary care interventions for e-cigarette use in teenagers and young adults. A case study of a 23 year old e-cigarette user shows promising results for tapering e-cigarette use with the assistance of a pharmacist[80], which suggests that different healthcare practitioners may play a role helping patients with gradually tapering off e-cigarettes. A randomized controlled trial of asthmatic teenagers who attended one of four clinics found that physicians discussed smoking during 38.2% of thee visits, but vaping was never brought up as a topic[81]. This emphasizes that physicians should discuss both smoking and vaping during appointments[81], in particular in youth presenting with asthma[74].

Medical curricula should stress that concurrent smoking and vaping screening and management interventions should be undertaken in the primary care setting. This way, many cases will not be missed given the high prevalence of dual use[50]. Family physicians should aim to identify youth at risk of vaping through screening questionnaires and aim to increase awareness of vaping for prevention purposes. This could include handing out

brochures to patients and their families about the health risks associated with vaping and therapies that are available which can assist with gradual tapering of nicotine from e-cigarettes. Family physicians have previously recommended open discussions with youth about risks during appointments[82] as well as educating families through public health educational campaigns[83].

It may also be strategic for medical, public health practitioners, and researchers to target particular groups and populations of teenagers that are most vulnerable to using e-cigarettes. A longitudinal study in the UK found an association between socioeconomic disadvantage and e-cigarette use in teenagers and young adults[84]. A systematic review also found that older teenagers from more affluent homes, of white ethnicity, and with higher levels of education had higher levels of knowledge and awareness of e-cigarette use, highlighting a possible need to educate younger teenagers with less education, ethnic minorities, and from lower income neighbourhoods [85]. It should be noted that one study found conflicting results with regards to the relationship between SES and e-cigarettes, whereby young adults from wealthier families were more likely to use e-cigarette, though the comparison groups were all in the affluent state of Connecticut[86].

Education was also found to be inversely associated with e-cigarette use in another study, but it had the greatest association in whites when compared with black young adults[87]. Vocational training, without higher education was found to also be associated with e-cigarette use in youth in Europe[88]. Thus, public health campaigns and medical doctors could potentially target individuals with lower levels of education, lower SES, and racially diverse groups to minimize any potential inequities in health.

Gender differences in e-cigarette use have also been noted in North America as well as Europe, whereby males were more likely to use them[88-90]. Additionally, since research indicates that females use e-cigarettes for mostly weight and stress management[91], interventions could focus on assisting them with stress along with making healthy lifestyle choices associated with weight.

Other particularly vulnerable groups have also included teenagers with impulsivity as well as those with mental health problems[92-94]. A study which explored EVALI cases found that mental health problems were prevalent in this population[94]. Thus, physicians and public health researchers may also consider screening and targeting individuals with mental health problems.

To date, there have been limited community-based and public health intervention trials to assist with e-cigarette prevention. "Catch my breath" was a prevention intervention in 12 middle schools across the U.S. The intervention focused on increasing knowledge on the harms associated with e-cigarette use[95]. The study authors found statistically significant differences in e-cigarette use prevalence in schools that had implemented the program when compared with control schools. They also found increased knowledge of e-cigarettes and the risks associated with their use[95].

Similarly, public health interventions targeting existing teenage users are in their infancy. There is a current text messaging intervention for e-cigarette cessation in teens in the United States[96]. The intervention provides users with educational content on e-cigarettes, focuses on fostering self-efficacy, assists with resilience building, and provides users with support and encouragement. The study had a very high enrolment after about one month of recruitment, with over 27,000 teenagers and young adults enrolled[96]. This indicates that this form of intervention is feasible, given the willingness for e-cigarette users to enrol [96]. Previous studies have found that text messaging for smoking cessation is effective and acceptable for this population[97-100], indicating that it could be used for vaping.

Additionally, there are very few commercially available e-cigarette cessation apps that can help teenagers and young adults quit. A systematic review of apps in the Google Play Store found that most apps encouraged e-cigarette use and that only 2 out of 79 were vaping cessation apps[101]. There is a need to develop an app that can be readily available and accessible to teenagers wanting to quit as well as an educational prevention app.

POLICIES

Strict policies to limit e-cigarette accessibility and exposure play an important role in preventing use. Research indicates that children and teenagers are exposed to e-cigarette marketing[102]. A study in the UK found that most e-cigarette advertisements were near children's stores and in areas that were less affluent[102], indicating that social health inequalities may exist, but more research is needed in this area. A review of 124 e-cigarette marketing publications revealed that companies have increased expenditures on social media campaigns and that they are often marketed as an alternative to cigarette smoking[103]. This is especially concerning given how social media may influence the decisions of teenagers and young adults. A randomized controlled trial found that by exposing youth without prior smoking history (N=417) to e-cigarette advertising (four advertisements), they were more likely to select e-cigarettes and have positive attitudes towards them relative to controls not exposed to this advertising[13]. Research had found that many e-cigarette advertisements on social media had used cartoons on packages to promote vaping in youth along with hashtags for vaping (#ejuice and #eliquid) [104]. The study authors also found that over 20% of advertisements had used a cartoon (66% of which were promotional posts), indicating that youth are often the targets of these ads across the globally accessible Instagram platform. They recommend similar policies to the ones for smoking including the Historical Master Settlement Agreement which banned advertising to youth[104]. Studies have also found that teenagers require multiple warnings in the forms of messages and ads to reduce their positive interest and susceptibility to e-cigarettes[105] and that perceptions of safety are related to environmental policy restrictions on vaping[106].

Research also indicates that patterns of e-cigarette use changed markedly in teenagers and young adults during the COVID-19 pandemic[107]. Changes in substance use behavioural patterns included ordering from alternative sellers, buying vaping products online, quitting vaping, and switching to cannabis or other products, resulting from the inherent challenges with making purchases at local vendors[107]. This emphasizes how the availability of vaping

products including their placement and immediate accessibility influences e-cigarette behavioural patterns including quitting[107].

Besides restricting marketing and advertisements, limiting the availability of e-cigarettes and accessibility to teenagers is greatly needed. A policy review on bans on the sale of e-cigarettes to minors across the United States found that e-cigarette use decreased along with smoking traditional cigarettes[108]. A qualitative study of adult vapers found that many agree with bans on advertising to minors to protect them[109].

A review of global vaping policies found that 68 countries regulate e-cigarettes and that the most frequent cross-national governmental policies include age limits (over 18 years of age), restricting advertisements, and placing bans on vaping in public places, while e-cigarette taxes are not commonly used[110]. The review found that Australia, the Czech Republic, and Malaysia classified e-cigarettes as toxic and poisonous substances[110]. Countries that have enacted child safety policies to protect children include Canada (banned flavoring and marketing to children)[111], Australia (available by prescription only with a child safety seal) [112] , New Zealand (banned vaping near schools) [113], the UK[110], the USA (some States have banned JUUL)[114], Finland, Germany, Ireland, Italy, Lithuania, Malta, Netherlands, and the Philippines[110]. Some countries with vape free restrictions that were also identified include France, Germany, Greece, Jamaica, Nepal, Portugal, Slovakia, Spain, Turkey, Venezuela, and Vietnam[110]. Countries with taxes on e-cigarettes include Italy, Latvia, Portugal, Republic of Korea, Togo, and the UK[110]. Asian countries that have banned e-cigarettes include Singapore and Thailand, and Japan has banned the use of nicotine containing e-cigarettes but not e-cigarettes without nicotine[115]. Vaping products are also prohibited in the United Arab Emirates[115]. Switzerland had banned the sale of vaping products until 2018, but now they are available on the market[116].

In developing countries, where resources are depleted and there is less regulatory oversight[117], concerns are raised about efforts to protect minors. Although data in India are limited, protective measures have nonetheless have been put into place in 2019, when e-cigarettes were banned to protect minors[118]. Concern has been raised in Guatemala over the lack of regulatory control over flavoured e-cigarettes which are enticing for teenagers[119]. While little is known about Africa, South Africa is planning on placing restrictions on e-cigarettes in 2021[120], but there has been strong opposition from the Tobacco Industry[121].

DISCUSSION

RECOMMENDATIONS

Without stricter interventions and policies, teenagers and young adult vapers will continue to be at risk of multiple health problems associated with e-cigarettes.
The following are a set of recommendations:
1. Strengthen global policies to restrict marketing, use of enticing flavouring, accessibility, and exposure to e-cigarettes in the environment

2. Increase physician education on screening and nicotine tapering in the primary care setting.
3. Increase public health education campaigns and develop evidence-based interventions.
4. Develop collaborations between physicians and public health researchers through joint efforts in education, screening, and referral.

Figure 5. illustrates strategies that may be applied from a social marketing perspective[122] to e-cigarettes by emphasizing that the health risks[40, 45, 50] should be reduced by restricting their access to children and teenagers[9], while the benefits of their use may be maximized when safely used in adult smokers attempting to quit[7]. It illustrates that screening, prevention, and intervention can take place in primary care settings and through public health interventions. Figure 6. illustrates a three-tiered approach to screening, education, prevention, and interventions for e-cigarettes in youth.

CONCLUSION

In summary, e-cigarettes pose a health threat to teenagers and young adults given the rise in the prevalence of use. While e-cigarettes are a safer alternative than smoking cigarettes and may be used as a harm reduction strategy in existing smokers, measures need to be urgently put into place to protect children and teenagers from unnecessary use and potential dual smoking and e-cigarette uptake. The outlook depends on whether sufficient primary care and public health strategies will be implemented to protect minors and young adults. As the long-term effects are unknown[61], it is especially prudent to limit unnecessary exposure. There is an urgent need to develop evidence-based primary care intervention and public health interventions that target vulnerable groups. Furthermore, there is need for stronger public health protection policies and bans to protect youth.

Table 1. Common Electronic Cigarette Related Terms[2-4, 36, 123, 124]

Term	Meaning
**Electronic Cigarettes**	A battery operated device, which heats a liquid, commonly containing glycerol, glycol, nicotine, flavourings, and other additives, producing an aerosol, which the user inhales with the mouthpiece. The cartridge is either refillable or disposable.
**E-Cigarettes/ E-Cigs**	Short terminology for electronic cigarettes
**Vape Pens**	Another term for e-cigarettes that are shaped like or resemble pens
**Pods**	Another term for e-cigarettes
**Vape Products**	A term which broadly includes all forms of electronic cigarettes made by different manufacturers including nicotine free e- cigarettes and ones with nicotine along with other chemical additives
**Vaping**	The act of (verb) using electronic cigarettes/ vape products by inhalation of the smoke free aerosol that is generated from the device
**Juice**	The liquid that is added to electronic cigarettes, which is heated for inhalation by the user. It can include nicotine, cannabis oil, flavourings, and other chemical additives.
**JUUL**	A discrete looking electronic cigarette device which resembles a USB drive and has a high content of nicotine
**EVALI**	Electronic Cigarette of Vaping Related Lung Injury resulting from the inhalation of vitamin E acetate and cannabis using electronic cigarettes

Table 2. PubMed/ Medline Search Strategy

Population

AND

"adolescent"[MeSH Terms] OR "adolescen*"[All Fields] OR "youth"[tiab] OR "youths"[tiab] OR "teen"[tiab] OR pubescen*[tiab] OR puberty[tiab] OR minor[tiab] OR minors[tiab] OR underage*[tiab] OR "under age*"[tiab] OR "young adult"[mesh] OR young adult*[tiab] OR "high school*"[tiab] OR student*[tiab]

Exposure:

AND

("electronic nicotine delivery systems"[MeSH Terms] OR "E cigarette*"[tiab] OR ecig*[tiab] OR "E Cig"[tiab] OR "E cigs"[tiab] OR "Electronic cigarette*"[tiab] OR Vaping[tiab] OR Vaper[tiab] OR Vapers[tiab] OR Vape[tiab] OR vapes[tiab] OR "Electronic nicotine"[tiab] OR "nicotine delivery"[tiab])

Intervention

AND

("Counseling"[Mesh] OR counsel*[tiab]) OR (((reduc*[ti] OR decreas*[ti]) AND (use[tiab] OR utilization[tiab])))) OR ("harm reduction*"[tiab] OR "Mass Screening"[Mesh] OR "screen*"[tiab] OR "routine testing"[tiab] OR experiment*[tiab] OR intervention*[tiab] OR study[tiab] OR studies[tiab] OR trial*[tiab] OR RCT[tiab] OR random*[tiab] OR "secondary prevention"[mesh] OR "tertiary prevention"[mesh] OR quit*[tiab] OR stop[ti] OR stopped[ti] OR stopping[ti] OR stops[ti])) OR ("Health campaign*"[tiab] OR "education campaign*"[tiab] OR "educational campaign*"[tiab] OR "media campaign*"[tiab])) OR (((Public health[ti] OR regulat*[ti]) AND (effect*[tiab] OR impact*[tiab] OR strateg*[tiab] OR campaign*[tiab] OR policy[tiab] OR policies[tiab] OR program*[tiab])))) OR (Taper*[tiab] OR "Behavioral support*"[tiab] OR "behavioural support*"[tiab] OR "Behavioral therap*"[tiab] OR "Behavioural therap*"[tiab] OR manag*[ti] OR control*[ti])) OR ((("flavoring agents"[mesh] OR flavor*[tiab] OR flavour*[tiab]) AND (restrict*[tiab] OR limit*[tiab] OR decreas*[tiab])))) OR ("reduced risk"[tiab] OR "risk reduction"[tiab] OR "vaping cessation"[tiab] OR "cessation therap*" OR "cessation treatment*"[tiab])) OR ((increas*[tiab] AND price[tiab]))) OR ("smoke free law"[tiab] OR "smoke free laws"[tiab] OR "population health standard*"[tiab] OR "warning label*"[tiab] OR tax[tiab] OR taxation[tiab] OR taxed[tiab] OR taxes[tiab] OR taxing[tiab] OR tax[tiab] OR taxation[tiab] OR taxed[tiab] OR taxes[tiab] OR taxing[tiab] OR "Commerce"[Mesh])) OR ((("Flavoring Agents"[Mesh] OR flavor*[tiab] OR flavour*[tiab]) AND (restricted[tiab] OR restriction[tiab] OR limit*[tiab] OR decreas*[tiab]) OR (increas*[tiab] AND price[tiab])))) OR (((reduc*[tiab] OR decreas*[tiab]) AND (use[tiab] OR utilization[tiab])))) OR ("Government Regulation"[Mesh] OR "Public Policy"[Mesh] OR "legislation and jurisprudence"[MeSH Subheading]) OR ("prevention and control"[sh] OR "public health"[mesh:noexp] OR "consumer product safety"[mesh] OR "public health practice"[mesh])

Outcomes

AND

"adverse effects" OR "Lung injury" OR "EVALI" OR "toxicity"[Subheading] OR "poisoning" OR "irritation" OR "inflammation" OR "pneumonia" OR "allergy" OR intoxication OR "respiratory effect" OR "cardiovascular" OR "health effect*" OR "case report*" OR "adverse effects"[Subheading] OR "lung injury"[MeSH Terms] OR "poisoning"[MeSH Terms] OR "poisoning"[Subheading] OR "poisons"[MeSH Terms] OR "poisons"[All Fields] OR "irritants"[MeSH Terms] OR "inflammation"[MeSH Terms] OR "pneumonia"[MeSH Terms] OR "hypersensitivity"[MeSH Terms] OR "allergy and immunology"[MeSH Terms] OR "cardiovascular system"[MeSH Terms] OR casereports[Filter]

Combined into one large search string

Figure 1. PRISMA Flow Chart

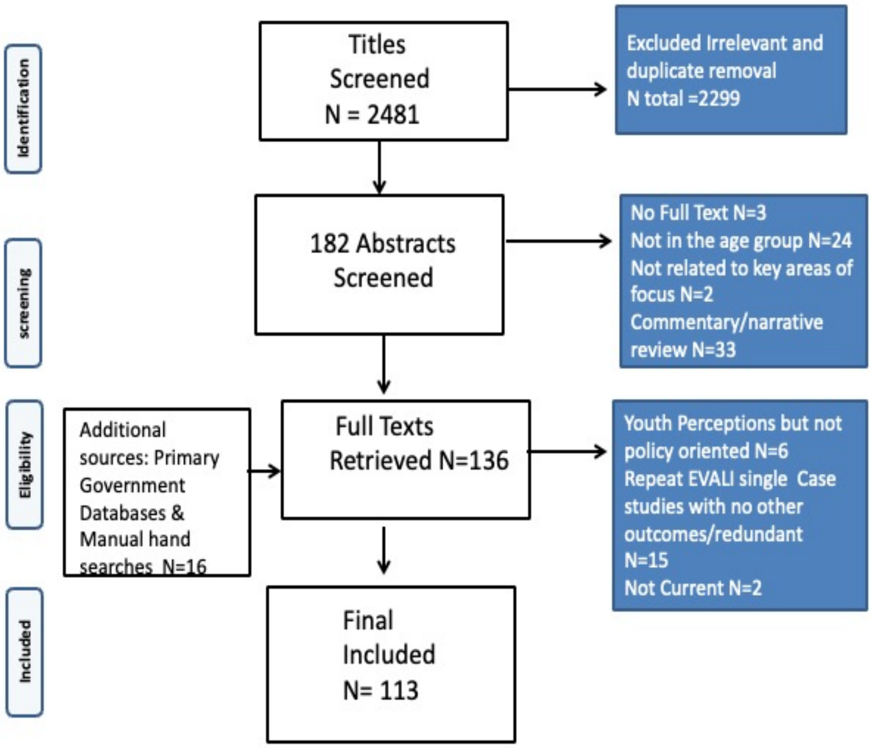

Table 3. E-Cigarettes Potential Harms and Benefits [6, 7, 34, 36, 45-47, 50, 61, 62, 71]

Exposure	Potential Benefits	Potential Risks
**E-Cigarettes**	• May assist with smoking cessation<br>• Potential harm reduction intervention for cigarette smokers as a form of nicotine replacement (also mimics smoking hand to mouth behaviours)<br>• Safer alternative than smoking cigarettes<br>• Less toxic chemicals and in lower doses than in conventional cigarettes	• Exposure of e-cigarettes to minors (children and teenagers) and previous non-smokers<br>• Potential gateway to smoking and initiation of other substances in teenagers<br>• Dual smoking and e-cigarette use compounds public health risks<br>• May increase the risk of respiratory health problems<br>• May increase the risk deficits in cognition, brain development, effort-reward imbalances in the brain in children and teenagers<br>• Long-term effects on health are unknown

Figure 2. History of "Ever Use" of E-Cigarettes in Teenagers by Country and Period[9, 17, 24-26, 29, 32]

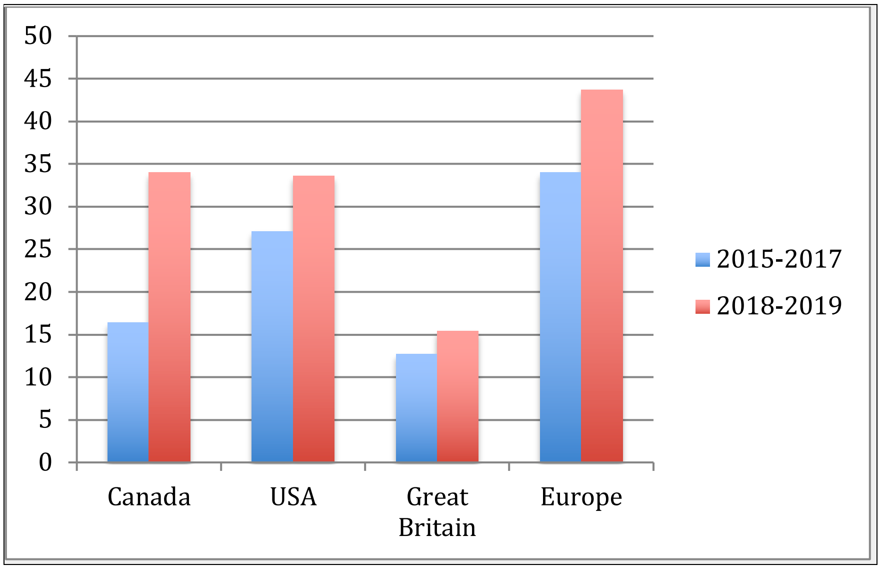

History of "ever use" of e-cigarettes in Canada, the USA, Great Britain, and the rest of Europe between 2015-2017 and 2018-2019[9, 17, 24-26, 29, 32]. If countries reported history of past use within any of these time periods, they were included. Please note that the 2015-2017 prevalence of ever use is for the following European countries: Belgium, Finland, Germany, Ireland, Italy, the Netherlands, and Portugal[27]. The 2018-2019 report in Europe collected data from Central and Eastern Europe including the following countries: Poland, Lithuania, Belarus, Slovakia, and Russia[17]. It should be noted that while the report by Hammond et al[9] reported a prevalence of "ever use" in the UK of 32.7% (2018), the Action and Smoking on Health Report in England[24] produced a significantly lower prevalence of 16.4% for the same period in Great Britain. It could be that Northern Ireland has a higher prevalence of ever use and was omitted from the report.

Figure 3. Prevalance of E-Cigarette Use (past 30 days) in Teenagers in North America Over a Five Year Period (between 2015-2020) [9, 26, 28-32]

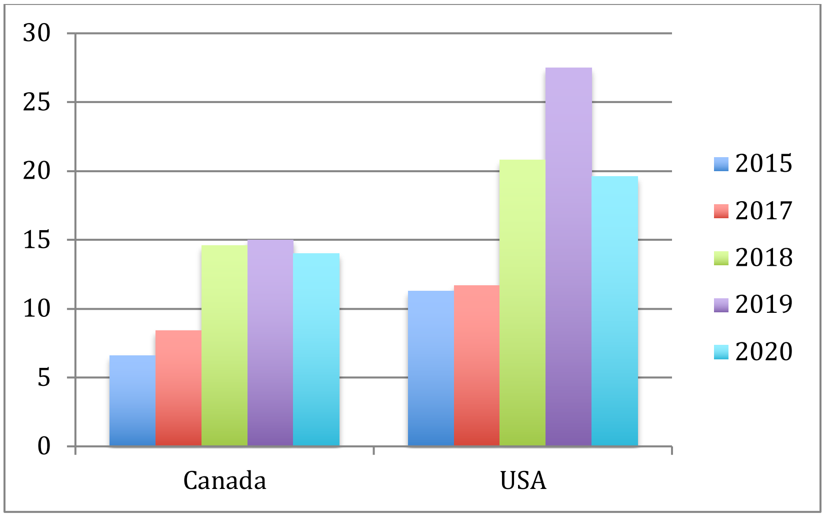

Past 30 day prevalence of e-cigarette use from 2015-2020 in North America (comparisons between Canada and the United States) in teenagers (grades 7 through 12). Reported prevalance declined in 2020 during the COVID-19 pandemic. The Canadian Tobacco and Nicotine Surveys were used for 2015, 2019, and 2020 surveys, respectively[26, 28]. The 2018-2019 surveys for Canada were obtained from the International Tobacco Control Policy Evaluation Project (ITC) Youth Tobacco and Vaping Survey, in Hammond et al[9]. The NYTS[29] (in the Surgeon's Report on E-Cigarette Usage), CDC, and FDA data from reports[30, 31] between 2015-2020 were also used. It should be noted that there is a slight discrepency in reported past 30 day prevalance of use in Canada between a Canadian report report in 2017[32] and the ITC survey report[9] (6.6% versus 8.4%) as well as between the FDA USA[31] report and the ITC in 2019[9] (20.8 versus 16.2%).

Figure 4. The health risks associated with vaping [21,22,29-31,34,43,51,53]

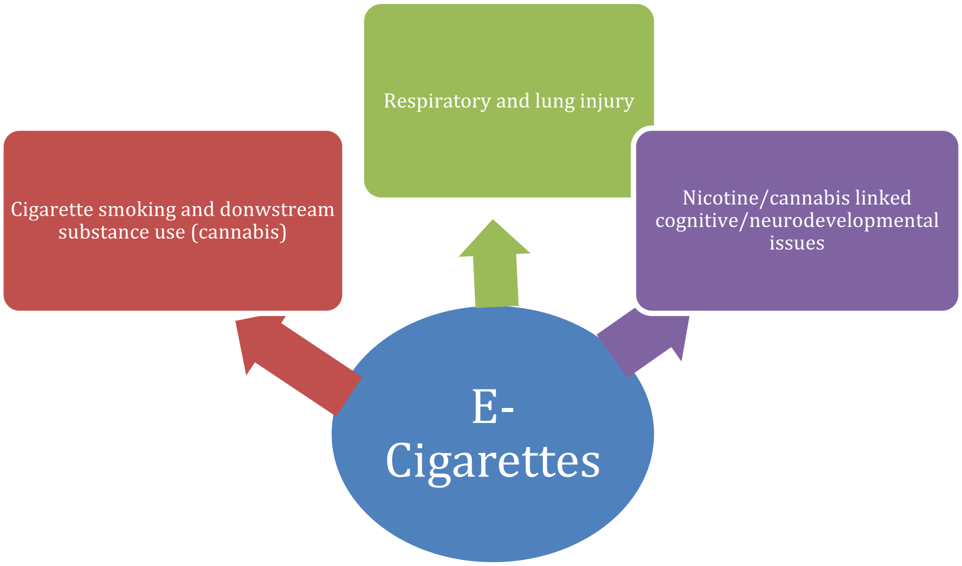

The following figure illustrates the relationship between e-cigarette exposure and potential health effects in teenagers, which primarily affect the respiratory system, neurodevelopment/ cognition, and may increase the risk of dual smoking and addiction to other drugs.

Figure 5. Strategies to reduce the harm and maximize the benefits associated with e-cigarettes at the individual and group levels [81,95-96,122]

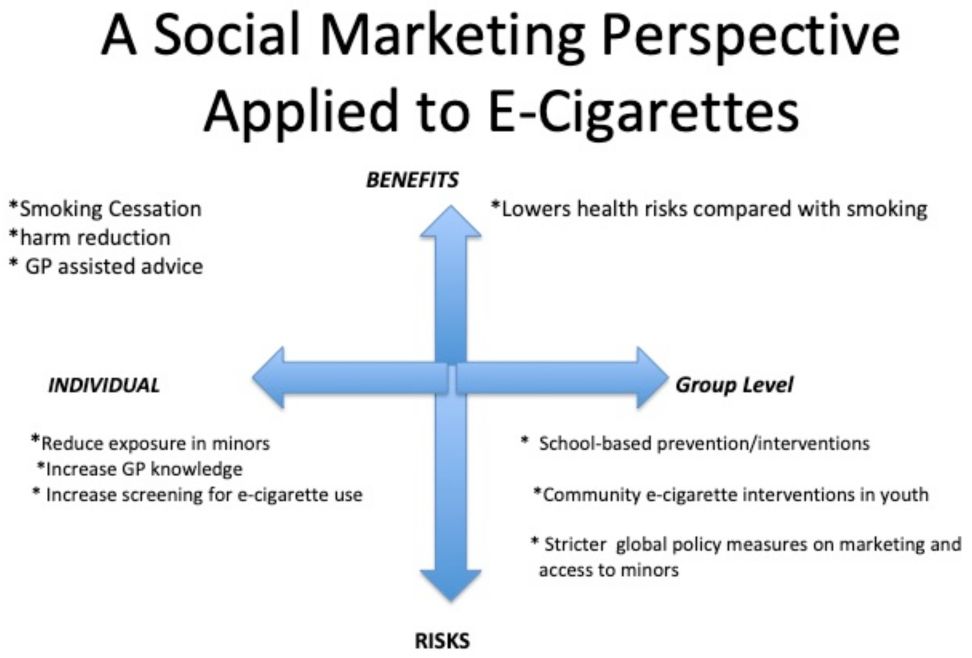

<u>Legend</u>

The North Axis represents the benefits of e-cigarettes for smokers and the South axis represents the risks, while the East and West axes represent the strategies that may be adopted at an individual level and community/population level. By maximizing the benefits in select adult smokers through harm reduction and minimizing the risks of exposure in minors, e-cigarettes may be safely used.

Figure 6. A three tiered approach to managing e-cigarette use in minors[125]

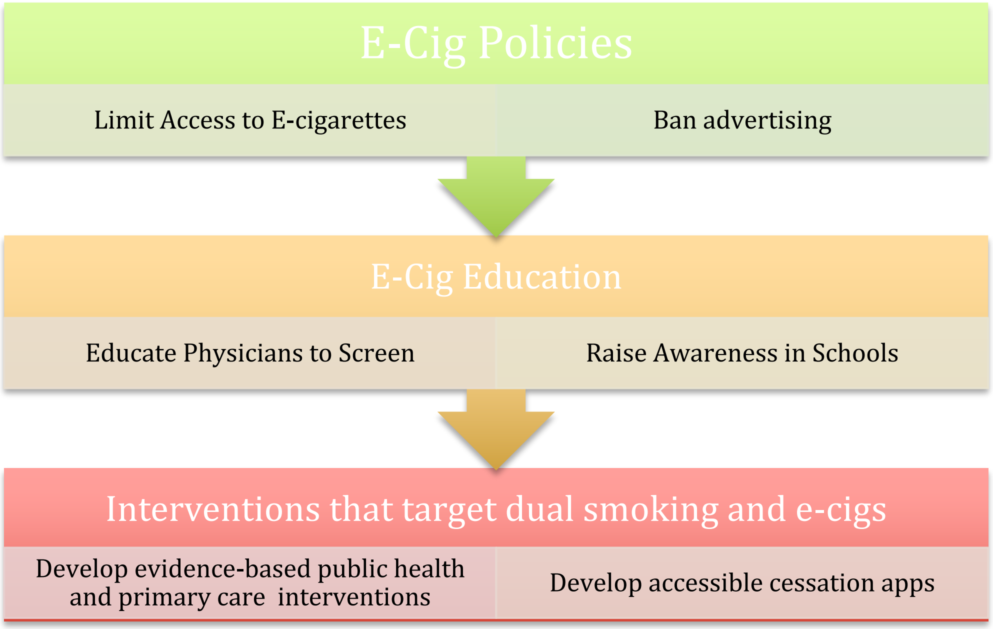

The following figure illustrates a three-tiered approach to managing e-cigarette use in minors which includes policy changes, awareness and prevention campaigns, and finally public health interventions that target existing teenage users.

References

1. Besaratinia A, Tommasi S. Vaping: A growing global health concern. *EClinicalMedicine*. Dec 2019;17:100208. doi:10.1016/j.eclinm.2019.10.019
2. Korfei M. The underestimated danger of E-cigarettes - also in the absence of nicotine. *Respiratory research*. Aug 29 2018;19(1):159. doi:10.1186/s12931-018-0870-4
3. Long GA. Comparison of select analytes in exhaled aerosol from e-cigarettes with exhaled smoke from a conventional cigarette and exhaled breaths. *International journal of environmental research and public health*. Oct 27 2014;11(11):11177-91. doi:10.3390/ijerph111111177
4. Hammond D, Wackowski OA, Reid JL, O'Connor RJ. Use of JUUL E-cigarettes Among Youth in the United States. *Nicotine & tobacco research : official journal of the Society for Research on Nicotine and Tobacco*. Apr 21 2020;22(5):827-832. doi:10.1093/ntr/nty237
5. Rahman MA, Hann N, Wilson A, Mnatzaganian G, Worrall-Carter L. E-cigarettes and smoking cessation: evidence from a systematic review and meta-analysis. *PloS one*. 2015;10(3):e0122544. doi:10.1371/journal.pone.0122544
6. Wang RJ, Bhadriraju S, Glantz SA. E-Cigarette Use and Adult Cigarette Smoking Cessation: A Meta-Analysis. *American journal of public health*. Feb 2021;111(2):230-246. doi:10.2105/ajph.2020.305999
7. Farsalinos KE, Polosa R. Safety evaluation and risk assessment of electronic cigarettes as tobacco cigarette substitutes: a systematic review. *Therapeutic advances in drug safety*. Apr 2014;5(2):67-86. doi:10.1177/2042098614524430
8. Barrington-Trimis JL, Urman R, Leventhal AM, et al. E-cigarettes, Cigarettes, and the Prevalence of Adolescent Tobacco Use. *Pediatrics*. Aug 2016;138(2)doi:10.1542/peds.2015-3983
9. Hammond D, Reid JL, Rynard VL, et al. Prevalence of vaping and smoking among adolescents in Canada, England, and the United States: repeat national cross sectional surveys. *BMJ (Clinical research ed)*. Jun 20 2019;365:l2219. doi:10.1136/bmj.l2219
10. Kong G, Morean ME, Cavallo DA, Camenga DR, Krishnan-Sarin S. Reasons for Electronic Cigarette Experimentation and Discontinuation Among Adolescents and Young Adults. *Nicotine & tobacco research : official journal of the Society for Research on Nicotine and Tobacco*. Jul 2015;17(7):847-54. doi:10.1093/ntr/ntu257
11. Morean ME, Butler ER, Bold KW, et al. Preferring more e-cigarette flavors is associated with e-cigarette use frequency among adolescents but not adults. *PloS one*. 2018;13(1):e0189015. doi:10.1371/journal.pone.0189015
12. Fadus MC, Smith TT, Squeglia LM. The rise of e-cigarettes, pod mod devices, and JUUL among youth: Factors influencing use, health implications, and

downstream effects. *Drug Alcohol Depend*. Aug 1 2019;201:85-93. doi:10.1016/j.drugalcdep.2019.04.011
13. Padon AA, Lochbuehler K, Maloney EK, Cappella JN. A Randomized Trial of the Effect of Youth Appealing E-Cigarette Advertising on Susceptibility to Use E-Cigarettes Among Youth. *Nicotine & tobacco research : official journal of the Society for Research on Nicotine and Tobacco*. Jul 9 2018;20(8):954-961. doi:10.1093/ntr/ntx155
14. Soneji SS, Knutzen KE, Villanti AC. Use of Flavored E-Cigarettes Among Adolescents, Young Adults, and Older Adults: Findings From the Population Assessment for Tobacco and Health Study. *Public health reports (Washington, DC : 1974)*. May/Jun 2019;134(3):282-292. doi:10.1177/0033354919830967
15. Perikleous EP, Steiropoulos P, Paraskakis E, Constantinidis TC, Nena E. E-Cigarette Use Among Adolescents: An Overview of the Literature and Future Perspectives. *Frontiers in public health*. 2018;6:86. doi:10.3389/fpubh.2018.00086
16. Government of Canada . Summary of results for the Canadian Student Tobacco, Alcohol and Drugs Survey 2018-19. *Retrieved September 2021 from:* https://www.canada.ca/en/health-canada/services/canadian-student-tobacco-alcohol-drugs-survey/2018-2019-summary.html
17. Brożek GM, Jankowski M, Lawson JA, et al. The Prevalence of Cigarette and E-cigarette Smoking Among Students in Central and Eastern Europe-Results of the YUPESS Study. *International journal of environmental research and public health*. Jun 28 2019;16(13)doi:10.3390/ijerph16132297
18. Kuwabara Y, Kinjo A, Fujii M, et al. Heat-not-burn tobacco, electronic cigarettes, and combustible cigarette use among Japanese adolescents: a nationwide population survey 2017. *BMC public health*. May 20 2020;20(1):741. doi:10.1186/s12889-020-08916-x
19. Lee JA, Lee S, Cho HJ. The Relation between Frequency of E-Cigarette Use and Frequency and Intensity of Cigarette Smoking among South Korean Adolescents. *International journal of environmental research and public health*. Mar 14 2017;14(3)doi:10.3390/ijerph14030305
20. Xiao L, Parascandola M, Wang C, Jiang Y. Perception and Current Use of E-cigarettes Among Youth in China. *Nicotine & tobacco research : official journal of the Society for Research on Nicotine and Tobacco*. Sep 19 2019;21(10):1401-1407. doi:10.1093/ntr/nty145
21. Oliveira WJC, Zobiole AF, Lima CB, et al. Electronic cigarette awareness and use among students at the Federal University of Mato Grosso, Brazil. *Jornal brasileiro de pneumologia : publicacao oficial da Sociedade Brasileira de Pneumologia e Tisilogia*. Set-Oct 2018;44(5):367-369. doi:10.1590/s1806-37562017000000229
22. Morello P, Pérez A, Peña L, et al. PREVALENCE AND PREDICTORS OF E-CIGARETTE TRIAL AMONG ADOLESCENTS IN ARGENTINA. *Tobacco prevention & cessation*. Dec 2016;2doi:10.18332/tpc/66950
23. Bhave SY, Chadi N. E-cigarettes and Vaping: A Global Risk for Adolescents. *Indian Pediatrics*. 2021/04/01 2021;58(4):315-319. doi:10.1007/s13312-021-2188-4
24. Action on Smoking and Health. Use of e-cigarettes among young people in

Great Britain. *Retrieved September 2021 from : https://ash.org.uk/wp-content/uploads/2021/07/Use-of-e-cigarettes-among-young-people-in-Great-Britain-2021.pdf*
25. Statistics Canada . Canadian Tobacco and Nicotine Survey 2016-2017. Retrieved May 2021 from: https://www150.statcan.gc.ca/n1/daily-quotidien/210317/dq210317b-eng.htm
26. Satistics Canada . Canadian Tobacco and Nicotine Survey 2018-2019. Retrieved May 2021 from : https://www150.statcan.gc.ca/n1/daily-quotidien/210317/dq210317b-eng.htm
27. Kinnunen JM, Rimpelä AH, Lindfors PL, et al. Electronic cigarette use among 14- to 17-year-olds in Europe. *Eur J Public Health*. Apr 24 2021;31(2):402-408. doi:10.1093/eurpub/ckaa145
28. Statistics Canada/ Canadian Tobacco and Nicotine Survey, 2016-2017. Retrieved May 2021 from: https://www23.statcan.gc.ca/imdb/p2SV.pl?Function=getSurvey&SDDS=5305
29. National Center for Chronic Disease Prevention and Health Promotion (US) Office on Smoking and Health. E-Cigarette Use Among Youth and Young Adults: A Report of the Surgeon General [Internet]. Atlanta (GA). *Centers for Disease Control and Prevention (US); 2016 Available from:* https://www.ncbi.nlm.nih.gov/books/NBK538680/
30. Centres for Disease Control (CDC). E-cigarette Use Among Middle and High School Students — United States, 2020. *Retrieved May 2021 from: https://www.cdc.gov/mmwr/volumes/69/wr/mm6937e1.htm*
31. FDA. 2018 NYTS Data: A Startling Rise in Youth E-cigarette Use. 2018. Retrieved May 2021 from: https://www.fda.gov/news-events/press-announcements/results-2018-national-youth-tobacco-survey-show-dramatic-increase-e-cigarette-use-among-youth-over
32. University of Waterloo. Tobacco Use in Canada. *2021. Retrieved from: https://uwaterloo.ca/tobacco-use-canada/e-cigarette-use-canada/prevalence-e-cigarette-use/e-cigarette-prevalence-age*
33. Kaur G, Pinkston R, McLemore B, Dorsey WC, Batra S. Immunological and toxicological risk assessment of e-cigarettes. *European respiratory review : an official journal of the European Respiratory Society*. Mar 31 2018;27(147)doi:10.1183/16000617.0119-2017
34. Tzortzi A, Kapetanstrataki M, Evangelopoulou V, Beghrakis P. A Systematic Literature Review of E-Cigarette-Related Illness and Injury: Not Just for the Respirologist. *International journal of environmental research and public health*. Mar 27 2020;17(7)doi:10.3390/ijerph17072248
35. Benyo SE, Bruinsma TJ, Drda E, et al. Risk Factors and Medical Symptoms Associated With Electronic Vapor Product Use Among Adolescents and Young Adults. *Clin Pediatr (Phila)*. Jun 2021;60(6-7):279-289. doi:10.1177/00099228211009681
36. Sun TWPLH. Vaping lung injuries top 1,000 cases as deaths rise to 18. Hundreds more people in the U.S. have been sickened by a mysterious vaping-related lung disease, and the death toll has risen to 18, according to federal health data released Thursday. 2019;

37. Blount BC, Karwowski MP, Shields PG, et al. Vitamin E Acetate in Bronchoalveolar-Lavage Fluid Associated with EVALI. *The New England journal of medicine*. Feb 20 2020;382(8):697-705. doi:10.1056/NEJMoa1916433
38. Abeles M, Popofsky S, Wen A, et al. Vaping-associated lung injury caused by inhalation of cannabis oil. *Pediatric pulmonology*. Jan 2020;55(1):226-228. doi:10.1002/ppul.24579
39. Boudi FB, Patel S, Boudi A, Chan C. Vitamin E Acetate as a Plausible Cause of Acute Vaping-related Illness. *Cureus*. Dec 11 2019;11(12):e6350. doi:10.7759/cureus.6350
40. Khan A, Parlette K, Kuntz HM. E-cigarettes and Vaping, Product-use Associated Lung Injury: A Case Series of Adolescents. *Clinical practice and cases in emergency medicine*. Feb 2021;5(1):11-16. doi:10.5811/cpcem.2020.10.48707
41. Gaiha SM, Cheng J, Halpern-Felsher B. Association Between Youth Smoking, Electronic Cigarette Use, and COVID-19. *The Journal of adolescent health : official publication of the Society for Adolescent Medicine*. Oct 2020;67(4):519-523. doi:10.1016/j.jadohealth.2020.07.002
42. Gotts JE, Jordt SE, McConnell R, Tarran R. What are the respiratory effects of e-cigarettes? *BMJ (Clinical research ed)*. Sep 30 2019;366:l5275. doi:10.1136/bmj.l5275
43. Gonzalez JE, Cooke WH. Acute effects of electronic cigarettes on arterial pressure and peripheral sympathetic activity in young nonsmokers. *Am J Physiol Heart Circ Physiol*. Jan 1 2021;320(1):H248-h255. doi:10.1152/ajpheart.00448.2020
44. Wang JB, Olgin JE, Nah G, et al. Cigarette and e-cigarette dual use and risk of cardiopulmonary symptoms in the Health eHeart Study. *PloS one*. 2018;13(7):e0198681. doi:10.1371/journal.pone.0198681
45. England LJ, Bunnell RE, Pechacek TF, Tong VT, McAfee TA. Nicotine and the Developing Human: A Neglected Element in the Electronic Cigarette Debate. *American journal of preventive medicine*. Aug 2015;49(2):286-93. doi:10.1016/j.amepre.2015.01.015
46. Goriounova NA, Mansvelder HD. Short- and long-term consequences of nicotine exposure during adolescence for prefrontal cortex neuronal network function. *Cold Spring Harbor perspectives in medicine*. Dec 1 2012;2(12):a012120. doi:10.1101/cshperspect.a012120
47. Yuan M, Cross SJ, Loughlin SE, Leslie FM. Nicotine and the adolescent brain. *The Journal of physiology*. Aug 15 2015;593(16):3397-412. doi:10.1113/jp270492
48. England LJ, Aagaard K, Bloch M, et al. Developmental toxicity of nicotine: A transdisciplinary synthesis and implications for emerging tobacco products. *Neuroscience and biobehavioral reviews*. Jan 2017;72:176-189. doi:10.1016/j.neubiorev.2016.11.013
49. Kim SI. Neuroscientific model of motivational process. *Frontiers in psychology*. 2013;4:98. doi:10.3389/fpsyg.2013.00098
50. Soneji S, Barrington-Trimis JL, Wills TA, et al. Association Between Initial Use of e-Cigarettes and Subsequent Cigarette Smoking Among Adolescents and Young Adults: A Systematic Review and Meta-analysis. *JAMA pediatrics*. Aug 1 2017;171(8):788-797. doi:10.1001/jamapediatrics.2017.1488

51. Dugas EN, Sylvestre MP, O'Loughlin J. Type of e-liquid vaped, poly-nicotine use and nicotine dependence symptoms in young adult e-cigarette users: a descriptive study. *BMC public health*. Jun 12 2020;20(1):922. doi:10.1186/s12889-020-09056-y
52. Aleyan S, Cole A, Qian W, Leatherdale ST. Risky business: a longitudinal study examining cigarette smoking initiation among susceptible and non-susceptible e-cigarette users in Canada. *BMJ open*. May 26 2018;8(5):e021080. doi:10.1136/bmjopen-2017-021080
53. Akre C, Suris JC. Adolescents and young adults' perceptions of electronic cigarettes as a gateway to smoking: a qualitative study in Switzerland. *Health education research*. Oct 1 2017;32(5):448-454. doi:10.1093/her/cyx054
54. Dai H, Catley D, Richter KP, Goggin K, Ellerbeck EF. Electronic Cigarettes and Future Marijuana Use: A Longitudinal Study. *Pediatrics*. May 2018;141(5)doi:10.1542/peds.2017-3787
55. Braymiller JL, Barrington-Trimis JL, Leventhal AM, et al. Assessment of Nicotine and Cannabis Vaping and Respiratory Symptoms in Young Adults. *JAMA network open*. Dec 1 2020;3(12):e2030189. doi:10.1001/jamanetworkopen.2020.30189
56. Noble MJ, Longstreet B, Hendrickson RG, Gerona R. Unintentional Pediatric Ingestion of Electronic Cigarette Nicotine Refill Liquid Necessitating Intubation. *Ann Emerg Med*. Jan 2017;69(1):94-97. doi:10.1016/j.annemergmed.2016.08.448
57. Lee LY, Burki NK, Gerhardstein DC, Gu Q, Kou YR, Xu J. Airway irritation and cough evoked by inhaled cigarette smoke: role of neuronal nicotinic acetylcholine receptors. *Pulmonary pharmacology & therapeutics*. 2007;20(4):355-64. doi:10.1016/j.pupt.2006.10.007
58. Vas CA, Porter A, McAdam K. Acetoin is a precursor to diacetyl in e-cigarette liquids. *Food and chemical toxicology : an international journal published for the British Industrial Biological Research Association*. Nov 2019;133:110727. doi:10.1016/j.fct.2019.110727
59. Son Y, Bhattarai C, Samburova V, Khlystov A. Carbonyls and Carbon Monoxide Emissions from Electronic Cigarettes Affected by Device Type and Use Patterns. *International journal of environmental research and public health*. Apr 17 2020;17(8)doi:10.3390/ijerph17082767
60. Meo SA, Ansary MA, Barayan FR, et al. Electronic Cigarettes: Impact on Lung Function and Fractional Exhaled Nitric Oxide Among Healthy Adults. *American journal of men's health*. Jan-Feb 2019;13(1):1557988318806073. doi:10.1177/1557988318806073
61. Bracken-Clarke D, Kapoor D, Baird AM, et al. Vaping and lung cancer - A review of current data and recommendations. *Lung cancer (Amsterdam, Netherlands)*. Mar 2021;153:11-20. doi:10.1016/j.lungcan.2020.12.030
62. Drummond MB, Upson D. Electronic cigarettes. Potential harms and benefits. *Annals of the American Thoracic Society*. Feb 2014;11(2):236-42. doi:10.1513/AnnalsATS.201311-391FR
63. Meo SA, Al Asiri SA. Effects of electronic cigarette smoking on human health. *European review for medical and pharmacological sciences*. 2014;18(21):3315-9.

64. Clapp PW, Pawlak EA, Lackey JT, et al. Flavored e-cigarette liquids and cinnamaldehyde impair respiratory innate immune cell function. *American journal of physiology Lung cellular and molecular physiology*. Aug 1 2017;313(2):L278-l292. doi:10.1152/ajplung.00452.2016
65. Muthumalage T, Lamb T, Friedman MR, Rahman I. E-cigarette flavored pods induce inflammation, epithelial barrier dysfunction, and DNA damage in lung epithelial cells and monocytes. *Scientific reports*. Dec 13 2019;9(1):19035. doi:10.1038/s41598-019-51643-6
66. O'Loughlin J, Lambert M, Karp I, et al. Association between cigarette smoking and C-reactive protein in a representative, population-based sample of adolescents. *Nicotine & tobacco research : official journal of the Society for Research on Nicotine and Tobacco*. Mar 2008;10(3):525-32. doi:10.1080/14622200801901997
67. Golbidi S, Edvinsson L, Laher I. Smoking and Endothelial Dysfunction. *Current vascular pharmacology*. 2020;18(1):1-11. doi:10.2174/1573403x14666180913120015
68. Boas Z, Gupta P, Moheimani RS, et al. Activation of the "Splenocardiac Axis" by electronic and tobacco cigarettes in otherwise healthy young adults. *Physiol Rep*. Sep 2017;5(17)doi:10.14814/phy2.13393
69. Tobore TO. On the potential harmful effects of E-Cigarettes (EC) on the developing brain: The relationship between vaping-induced oxidative stress and adolescent/young adults social maladjustment. *Journal of adolescence*. Oct 2019;76:202-209. doi:10.1016/j.adolescence.2019.09.004
70. Dai H, Hao J. Electronic cigarette and marijuana use among youth in the United States. *Addictive behaviors*. Mar 2017;66:48-54. doi:10.1016/j.addbeh.2016.11.005
71. Ren M, Lotfipour S. Nicotine Gateway Effects on Adolescent Substance Use. *The western journal of emergency medicine*. Aug 20 2019;20(5):696-709. doi:10.5811/westjem.2019.7.41661
72. Wong DN, Fan W. Ethnic and sex differences in E-cigarette use and relation to alcohol use in California adolescents: the California Health Interview Survey. *Public health*. Apr 2018;157:147-152. doi:10.1016/j.puhe.2018.01.019
73. National Academies of Sciences E, and Medicine; Health and Medicine Division; Board on Population Health and Public Health Practice; Committee on the Review of the Health Effects of Electronic Nicotine Delivery Systems. Editors: David L. Eaton, Leslie Y. Kwan, and Kathleen Stratton. Public Health Consequences of E-Cigarettes.
Washington (DC): National Academies Press (US); 2018 Jan 23
ISBN-13: 978-0-309-46834-3ISBN-10: 0-309-46834-5. 2018;
74. Bayly JE, Bernat D, Porter L, Choi K. Secondhand Exposure to Aerosols From Electronic Nicotine Delivery Systems and Asthma Exacerbations Among Youth With Asthma. *Chest*. Jan 2019;155(1):88-93. doi:10.1016/j.chest.2018.10.005
75. Alnajem A, Redha A, Alroumi D, et al. Use of electronic cigarettes and secondhand exposure to their aerosols are associated with asthma symptoms among adolescents: a cross-sectional study. *Respir Res*. Nov 16 2020;21(1):300. doi:10.1186/s12931-020-01569-9

76. Pepper JK, Gilkey MB, Brewer NT. Physicians' Counseling of Adolescents Regarding E-Cigarette Use. *The Journal of adolescent health : official publication of the Society for Adolescent Medicine*. Dec 2015;57(6):580-6. doi:10.1016/j.jadohealth.2015.06.017
77. El-Shahawy O, Brown R, Elston Lafata J. Primary Care Physicians' Beliefs and Practices Regarding E-Cigarette Use by Patients Who Smoke: A Qualitative Assessment. *International journal of environmental research and public health*. Apr 26 2016;13(5)doi:10.3390/ijerph13050445
78. Abadi S, Couch ET, Chaffee BW, Walsh MM. Perceptions Related to Use of Electronic Cigarettes among California College Students. *J Dent Hyg*. Feb 2017;91(1):35-43.
79. Geletko KW, Myers K, Brownstein N, et al. Medical Residents' and Practicing Physicians' e-Cigarette Knowledge and Patient Screening Activities: Do They Differ? *Health services research and managerial epidemiology*. Jan-Dec 2016;3:2333392816678493. doi:10.1177/2333392816678493
80. Sahr M, Kelsh SE, Blower N. Pharmacist assisted vape taper and behavioral support for cessation of electronic nicotine delivery system use. *Clinical case reports*. Jan 2020;8(1):100-103. doi:10.1002/ccr3.2599
81. Beznos B, Sayner R, Carpenter DM, et al. Communication About Adolescent and Caregiver Smoking and Vaping During Pediatric Asthma Visits: Implications for Providers. *J Pediatr Health Care*. Jul-Aug 2021;35(4):401-407. doi:10.1016/j.pedhc.2021.02.004
82. Arane K, Goldman RD. Electronic cigarettes and adolescents. *Can Fam Physician*. Nov 2016;62(11):897-898.
83. Alexander JP, Williams P, Lee YO. Youth who use e-cigarettes regularly: A qualitative study of behavior, attitudes, and familial norms. *Prev Med Rep*. Mar 2019;13:93-97. doi:10.1016/j.pmedr.2018.11.011
84. Green MJ, Gray L, Sweeting H, Benzeval M. Socioeconomic patterning of vaping by smoking status among UK adults and youth. *BMC public health*. Feb 10 2020;20(1):183. doi:10.1186/s12889-020-8270-3
85. Hartwell G, Thomas S, Egan M, Gilmore A, Petticrew M. E-cigarettes and equity: a systematic review of differences in awareness and use between sociodemographic groups. *Tobacco control*. Dec 2017;26(e2):e85-e91. doi:10.1136/tobaccocontrol-2016-053222
86. Simon P, Camenga DR, Morean ME, et al. Socioeconomic status and adolescent e-cigarette use: The mediating role of e-cigarette advertisement exposure. *Preventive medicine*. Jul 2018;112:193-198. doi:10.1016/j.ypmed.2018.04.019
87. Assari S, Mistry R, Bazargan M. Race, Educational Attainment, and E-Cigarette Use. *Journal of medical research and innovation*. 2020;4(1)doi:10.32892/jmri.185
88. Surís JC, Berchtold A, Akre C. Reasons to use e-cigarettes and associations with other substances among adolescents in Switzerland. *Drug and alcohol dependence*. Aug 1 2015;153:140-4. doi:10.1016/j.drugalcdep.2015.05.034
89. Cooper M, Case KR, Loukas A, Creamer MR, Perry CL. E-cigarette Dual Users, Exclusive Users and Perceptions of Tobacco Products. *American journal of health behavior*. Jan 2016;40(1):108-16. doi:10.5993/ajhb.40.1.12

90. Hanewinkel R, Isensee B. Risk factors for e-cigarette, conventional cigarette, and dual use in German adolescents: a cohort study. *Preventive medicine*. May 2015;74:59-62. doi:10.1016/j.ypmed.2015.03.006
91. Piñeiro B, Correa JB, Simmons VN, et al. Gender differences in use and expectancies of e-cigarettes: Online survey results. *Addictive behaviors*. Jan 2016;52:91-7. doi:10.1016/j.addbeh.2015.09.006
92. Bold KW, Morean ME, Kong G, et al. Early age of e-cigarette use onset mediates the association between impulsivity and e-cigarette use frequency in youth. *Drug and alcohol dependence*. Dec 1 2017;181:146-151. doi:10.1016/j.drugalcdep.2017.09.025
93. Alanazi AMM, Alqahtani MM, Pavela G, Ford EW, Leventhal AM, Hendricks PS. Mental Health and the Association between Asthma and E-cigarette Use among Young Adults in The United States: A Mediation Analysis. *International journal of environmental research and public health*. Nov 26 2020;17(23)doi:10.3390/ijerph17238799
94. Adkins SH, Anderson KN, Goodman AB, et al. Demographics, Substance Use Behaviors, and Clinical Characteristics of Adolescents With e-Cigarette, or Vaping, Product Use-Associated Lung Injury (EVALI) in the United States in 2019. *JAMA pediatrics*. Jul 1 2020;174(7):e200756. doi:10.1001/jamapediatrics.2020.0756
95. Kelder SH, Mantey DS, Van Dusen D, Case K, Haas A, Springer AE. A Middle School Program to Prevent E-Cigarette Use: A Pilot Study of "CATCH My Breath". *Public health reports (Washington, DC : 1974)*. Mar/Apr 2020;135(2):220-229. doi:10.1177/0033354919900887
96. Graham AL, Jacobs MA, Amato MS. Engagement and 3-Month Outcomes From a Digital E-Cigarette Cessation Program in a Cohort of 27 000 Teens and Young Adults. *Nicotine & tobacco research : official journal of the Society for Research on Nicotine and Tobacco*. Apr 21 2020;22(5):859-860. doi:10.1093/ntr/ntz097
97. Haug S, Schaub MP, Venzin V, Meyer C, John U. Efficacy of a text message-based smoking cessation intervention for young people: a cluster randomized controlled trial. *J Med Internet Res*. Aug 16 2013;15(8):e171. doi:10.2196/jmir.2636
98. McClure E, Baker N, Carpenter MJ, Treiber FA, Gray K. Attitudes and interest in technology-based treatment and the remote monitoring of smoking among adolescents and emerging adults. *Journal of smoking cessation*. Jun 2017;12(2):88-98. doi:10.1017/jsc.2015.15
99. Müssener U, Bendtsen M, McCambridge J, Bendtsen P. User satisfaction with the structure and content of the NEXit intervention, a text messaging-based smoking cessation programme. *BMC public health*. Nov 22 2016;16(1):1179. doi:10.1186/s12889-016-3848-5
100. Müssener U, Bendtsen M, Karlsson N, White IR, McCambridge J, Bendtsen P. Effectiveness of Short Message Service Text-Based Smoking Cessation Intervention Among University Students: A Randomized Clinical Trial. *JAMA internal medicine*. Mar 2016;176(3):321-8. doi:10.1001/jamainternmed.2015.8260
101. Meacham MC, Vogel EA, Thrul J. Vaping-Related Mobile Apps Available in the Google Play Store After the Apple Ban: Content Review. *Journal of medical Internet research*. Nov 13 2020;22(11):e20009. doi:10.2196/20009

102. Eadie D, Stead M, MacKintosh AM, et al. E-cigarette marketing in UK stores: an observational audit and retailers' views. *BMJ open*. Sep 11 2015;5(9):e008547. doi:10.1136/bmjopen-2015-008547
103. Collins L, Glasser AM, Abudayyeh H, Pearson JL, Villanti AC. E-Cigarette Marketing and Communication: How E-Cigarette Companies Market E-Cigarettes and the Public Engages with E-cigarette Information. *Nicotine & tobacco research : official journal of the Society for Research on Nicotine and Tobacco*. Jan 1 2019;21(1):14-24. doi:10.1093/ntr/ntx284
104. Allem JP, Cruz TB, Unger JB, Toruno R, Herrera J, Kirkpatrick MG. Return of cartoon to market e-cigarette-related products. *Tobacco control*. Sep 2019;28(5):555-557. doi:10.1136/tobaccocontrol-2018-054437
105. Andrews JC, Mays D, Netemeyer RG, Burton S, Kees J. Effects of E-Cigarette Health Warnings and Modified Risk Ad Claims on Adolescent E-Cigarette Craving and Susceptibility. *Nicotine & tobacco research : official journal of the Society for Research on Nicotine and Tobacco*. May 21 2019;21(6):792-798. doi:10.1093/ntr/nty076
106. Agaku IT, Perks SN, Odani S, Glover-Kudon R. Associations between public e-cigarette use and tobacco-related social norms among youth. *Tobacco control*. May 2020;29(3):332-340. doi:10.1136/tobaccocontrol-2018-054728
107. Gaiha SM, Lempert LK, Halpern-Felsher B. Underage Youth and Young Adult e-Cigarette Use and Access Before and During the Coronavirus Disease 2019 Pandemic. *JAMA network open*. Dec 1 2020;3(12):e2027572. doi:10.1001/jamanetworkopen.2020.27572
108. Abouk R, Adams S. Bans on electronic cigarette sales to minors and smoking among high school students. *Journal of health economics*. Jul 2017;54:17-24. doi:10.1016/j.jhealeco.2017.03.003
109. Farrimond H. E-cigarette regulation and policy: UK vapers' perspectives. *Addiction (Abingdon, England)*. Jun 2016;111(6):1077-83. doi:10.1111/add.13322
110. Kennedy RD, Awopegba A, De León E, Cohen JE. Global approaches to regulating electronic cigarettes. *Tobacco control*. Jul 2017;26(4):440-445. doi:10.1136/tobaccocontrol-2016-053179
111. Government of Canada. Vaping Products – New limits on nicotine concentration and consultation on flavour restrictions. Retrieved September 2021 from: https://www.canada.ca/en/health-canada/news/2021/06/backgrounder-vaping-products--new-limits-on-nicotine-concentration-and-consultation-on-flavour-restrictions.html
112. Australian Government. Therapeutic Goods Administration. Nicotine e-cigarettes laws are changing. *Retrieved September 2021 from: https://www.tga.gov.au/blogs/tga-topics/nicotine-vaping-laws-are-changing*
113. New Zealand. Ministry of Health. Regulation of vaping and smokeless tobacco products. *Retrieved Sept 2021 from: https://www.health.govt.nz/our-work/regulation-health-and-disability-system/regulation-vaping-and-smokeless-tobacco-products*

114. Ducharme, Jamie. As the Number of Vaping-Related Deaths Climbs, These States Have Implemented E-Cigarette Bans. *Time*. 2019. Retrieved Sept 2021 from: https://time.com/5685936/state-vaping-bans/
115. Doan TTT, Tan KW, Dickens BSL, Lean YA, Yang Q, Cook AR. Evaluating smoking control policies in the e-cigarette era: a modelling study. *Tobacco control*. Sep 2020;29(5):522-530. doi:10.1136/tobaccocontrol-2019-054951
116. Swiss Info. Swiss Court overturns Ban on Vaping Products. *2018. retrieved September 2021 from: https://www.swissinfo.ch/eng/business/immediate-effect_court-overturns-swiss-ban-on-e-cigarettes/44082174*
117. Otañez MG, Mamudu HM, Glantz SA. Tobacco companies' use of developing countries' economic reliance on tobacco to lobby against global tobacco control: the case of Malawi. *American journal of public health*. Oct 2009;99(10):1759-71. doi:10.2105/ajph.2008.146217
118. Chakma JK, Kumar H, Bhargava S, Khanna T. The e-cigarettes ban in India: an important public health decision. *The Lancet Public health*. Aug 2020;5(8):e426. doi:10.1016/s2468-2667(20)30063-3
119. Monzón J, Islam F, Mus S, Thrasher JF, Barnoya J. Effects of tobacco product type and characteristics on appeal and perceived harm: Results from a discrete choice experiment among Guatemalan adolescents. *Preventive medicine*. Jul 2021;148:106590. doi:10.1016/j.ypmed.2021.106590
120. BusinessTech. Push for new e-cigarette and smoking laws in South Africa. *BusinessTech*. 2021. retrieved September 2021 from: https://businesstech.co.za/news/lifestyle/472460/push-for-new-e-cigarette-and-smoking-laws-in-south-africa/
121. BusinessTech. Tobacco industry pushes back against new smoking and vaping laws for South Africa. *BusinessTech*. 2021. Retrieved September 2021 from: https://businesstech.co.za/news/lifestyle/491565/tobacco-industry-pushes-back-against-new-smoking-and-vaping-laws-for-south-africa/
122. The National Social Marketing Centre NSCM. Social Marketing Benchmark Criteria. *NSMC*. 2008. Retrieved September 2021 from: https://www.thensmc.com/sites/default/files/benchmark-criteria-090910.pdf
123. Johns Hopkins Medicine . Vape Flavours and Vape juice: What you need to know. *Hopkins Medicine*. 2021. Retrieved September 2021 from: https://www.hopkinsmedicine.org/health/wellness-and-prevention/vape-flavors-and-vape-juice-what-you-need-to-know
124. CDC. E-cigarettes, Or Vaping Products Visual Dictionary. 2021. Retrieved September 2021 from: https://www.cdc.gov/tobacco/basic_information/e-cigarettes/pdfs/ecigarette-or-vaping-products-visual-dictionary-508.pdf
125. Lyzwinski LN, Eisenberg, M. Vaping and Polysubstance Abuse in Teenagers and Young Adults: The Rise in Cannabis and Nicotine Co-Use With E-Cigarettes and Associated Health Risks. Chapter In The Handbook of Substance Misuse and Addictions: From Biology to Public Health, edited by Vinood B. Patel and Victor R. Preedy. *Nature Springer*. 2021. In press.
