# Supplementary material for: Global youth vaping and respiratory health: epidemiology, interventions, and policies
Source: NPJ Prim Care Respir Med. 2022 Apr 11;32:14. doi: 10.1038/s41533-022-00277-9 (PMC9001701; doi:10.1038/s41533-022-00277-9)
Supplement: Supplementary file 3 — Checklist for NPJRM [file 41533_2022_277_MOESM3_ESM.pdf]

MANUSCRIPT CHECKLIST

	YES	N/A
**TITLE**	X	
Should be no more than 150 characters	X	
Remove all claims of primacy, such as ‘novel’ or ‘first’	X	
The title should be on the first page of the manuscript and should be the same as the title in the online submission system.	x	
**AUTHORSHIP**		
Check that the authorship list and affiliations on the title page of the manuscript are the same as the online submission system (corresponding author will be listed first in the online submission system)	X	
Funding sources should be disclosed in the Acknowledgements section.	X	
Include an Author Contributions section after the Competing Interests section. All authors should meet all four criteria: 1) Substantial contributions to the conception or design of the work or the acquisition, analysis or interpretation of the data, 2) Drafting the work or revising it critically for important intellectual content, 3) Final approval of the completed version, 4) Accountability for all aspects of the work in ensuring that questions related to the accuracy or integrity of any part of the work are appropriately investigated and resolved	x	
If multiple authors are considered “co-first author”, please include a statement in the Author Contributions section.		N/A
Any authorship changes (additions, removals, spelling changes, or re-ordering) should be accompanied by an authorship change form signed by all authors, including those who will be removed	X	
Ensure that the corresponding author(s) are clearly marked, and that an email address is given for each	x	
**ABSTRACT**		
Should be no more than 250 words	X	
Should not contain references	X	
Remove all claims of primacy, such as ‘novel’ or ‘first’	X	
Check that the abstract in the manuscript is the same as the abstract in the online submission system	x	
**MAIN TEXT**		
Should be provided as a Word or Tex document	X	
Double-spaced, single-column without justification and no footnotes	X	
Pages numbered using Arabic numerals in the footer of each page	X	
Section order for original research articles and brief communications: Title page, Abstract and Keywords, Introduction, Results, Discussion, Methods, Data Availability Statement, Acknowledgements, Competing Interests, Author Contributions, References, Figure Legends	X	
Should not contain tables or figures. Tables and figures should be uploaded as individual files (1 file per table or figure)	x	
Equations should be labeled with Arabic numerals		X
Subheadings can be used and should be in bold	X	

Data Availability Statement: All manuscripts must include this as a separate section at the end of the main text. Refer to the Guide to Authors for more information about this policy and examples.	X	
Competing Interests: All competing interests must be declared, not limited to funding, employment, financial etc. Refer to our Guide to Authors for details.	x	
Remove all claims of primacy, such as ‘novel’ and ‘first’	X	
For all human and animal studies, include a statement in the Methods section naming the agency or committee that granted approval (see “studies using human subjects” checklist section below)		x
**REFERENCES**		
Should be cited in correct numerical order	x	
Should contain only 1 reference per number and each reference should only be listed once	x	
Should include only published work or work “in press”	X	
References should list up to 5 authors. If a reference has more than 5 authors, include the first author followed by *et al.*	x	
References should be formatted in the following Nature style: Authors, Title, Journal, Volume, First-last page, (year)	x	
References to web-only journals or websites: Authors, Title, Journal (if a journal), url/doi, (year)	x	
References to preprint servers: Authors.Preprint title. Preprint at [link to preprint] (year)	x	
**FIGURE LEGENDS**		
Should be included as the last section in the manuscript text	X	
Contain a brief title and every figure panel should be described	X	
Length of scale bars should be defined in the figure legend, not in the figure		N/A
Error bars should be defined as standard deviation (s.d.) or standard error of the mean (s.e.m)		X
**FIGURES**		
Each figure should be uploaded as its own file (not within the manuscript text file)	X	
Figure panels should be arranged into rectangular shapes	x	
Each panel should be labeled with a single lowercase letter and panels should not be subdivided.	x	
Scale bars should be included but not labeled within the figure.	x	
Blots and gels should contain molecular weight or size markers and loading controls.		x
Blots and gels must include a statement in the methods section or relevant figure legends that all blots or gels derive from the same experiment and that they were processed in parallel.		x
Axes of graphs should be labeled and include units.	X	
Check whether the manuscript contains third-party images, such as figures from the literature, stock photos, clip art or commercial satellite and map data. We strongly discourage the use or adaptation of previously published images, but if this is unavoidable, please request the necessary license to re-use such material from the relevant copyright holders and return this to us when you submit your revised manuscript. The attribution given in the license must be included, and the reference for the citation given.		X
**TABLES**		
Each table should be uploaded as its own file (not within the manuscript text file).	X	

Should contain at least 2 columns with heading for each column.	X	
Should be contained within Word or Tex files	X	
Include a title without punctuation	X	
References to table footnotes should be made with Arabic numerals	X	
**SUPPLEMENTARY FILES**		N/A
Check the merged PDF and delete any duplicate manuscript, figure or table files		
Supplementary files should be less than 30 MB		
Supplementary files should be labeled and contain only these elements: Supplementary Figures with legends beneath each one / Supplementary Tables / Supplementary notes /Supplementary Discussion / Supplementary Methods / Supplementary References		
Each supplementary item should be cited in the main manuscript text		
Supplementary items are not confidential. If you need to upload confidential files for the Editor or Reviewers, please inform the journal staff and include “confidential” in the file title.	X	
Proofs of the supplementary material will not be provided and will appear close to how they appear on the system. Please check thoroughly that it appears as you would like to see published.		
**STUDIES INVOLVING HUMAN SUBJECTS**		N/A
Reporting Summary checklist (for all life science research)		x
Editorial Policy checklist (for all life science research)		
Include a statement about written informed consent in the Methods section (for all human studies)		x
Remove or limit patient identifiers within the text, figures, tables, and supplementary material (for all human studies). All reasonable measures must be taken to protect patient anonymity. Black bars over the eyes are not acceptable means of anonymization. When including identifiable images or data from human research participants, please include a statement in the manuscript text affirming that you have obtained informed consent for the specific images or data. Images without appropriate consent should not be included in the manuscript.		x
For patient photographs, provide a consent form for any photographs of human subjects and include statements in the Methods section as well as corresponding figure legend that written consent was obtained for publication of the photographs.		X
For clinical trials, include the clinical trial registration number in the online submission system as well as in the manuscript text.		X
For clinical trials, attach the clinical trial protocol as a separate document.		X
For randomized clinical trials, submit a CONSORT checklist.		X

Additional information can be found in the Guide to Authors.
