# Supplementary material for: Global youth vaping and respiratory health: epidemiology, interventions, and policies
Source: NPJ Prim Care Respir Med. 2022 Apr 11;32:14. doi: 10.1038/s41533-022-00277-9 (PMC9001701; doi:10.1038/s41533-022-00277-9)
Supplement: Supplementary file 1 — original submission cover letter copy [file 41533_2022_277_MOESM1_ESM.pdf]

Dear Dr. Tsiligianni,

I corresponded with Dr Viki Hurst at Nature who contacted you directly. Many thanks for inviting our review for submission.

I have attached the previous correspondence here about inviting our review for submission to Primary Care Respiratory Medicine (npj). We have now updated the paper according to the recommendations. I am submitting this as a scoping review, but I also have a version B. Perspective on file as well.

We believe that this paper is important given the global vaping problem among teenagers and young adults. We also believe that this is a thorough review which comprehensively covers important dimensions of the problem including epidemiological trends, mechanisms, policies, interventions, and recommendations for future health promotion.

Thank You.

Sincerely,

Lynnette Lyzwinski, PhD, MPhil PH
McGill University, Faculty of Medicine

From: **Viki Hurst** viki.hurst@nature.com
Subject: RE: Call for submission Respiratory Medicine Query
Date: September 20, 2021 at 6:34 PM
To: Lynnette Nathalie Lyzwinski lynnettenathalie@icloud.com

VH

Dear Lynnette,

Thank you for your patience with this! I have now obtained some feedback from the Chief Editor on your paper. Dr Tsiligianni indicated that she would welcome the full submission at *npj Primary Care Respiratory Medicine*, with the following points addressed:

1. This submission should either mention that it is a scoping review, or you could consider sending it as a Perspective.
2. The discussion about the interconnection of public health with primary care should be strengthened, and should include suggestions for improvement.

Please do mention this consultation in your cover letter, and I will bring it to Dr Tsiligianni's attention when we have received this in the system. You can submit your manuscript here: https://mts-npjpcrm.nature.com/cgi-bin/main.plex

Best wishes,

Viki

From: Lynnette Nathalie Lyzwinski <lynnettenathalie@icloud.com>
Sent: 14 September 2021 15:22
To: Viki Hurst <viki.hurst@nature.com>
Subject: Re: Call for submission Respiratory Medicine Query

[External - Use Caution]

Dear Dr Hurst,
hope you're having a nice week!
We were just wondering if the Editor in Chief might be interested in our paper?
I have attached it here again.
Many thanks.
Best,
Lynnette

> On Aug 27, 2021, at 3:17 PM, Viki Hurst <viki.hurst@nature.com> wrote:
>
> Dear Lynnette,
>
> Thank you for sending this presubmission inquiry to npj Primary Care Respiratory Medicine.
>
> I just wanted to let you know that it has been forwarded to our Editor in Chief of the journal for her assessment, however she is currently on annual leave and has not been picking up emails.
>
> Please do contact me at the end of the next week if you have not received any feedback and I will request this for you.
